# Copper-based semiconductor nanocrystals for optical applications

**DOI:** 10.1039/d5nr04617c

**Published:** 2026-03-13

**Authors:** Riccardo Marin, Lucas V. Besteiro, Patrizia Canton

**Affiliations:** a Intelligent Optical Nanomaterials (IONs) group, Dipartimento di Scienze Molecolari e Nanosistemi, Università Ca’ Foscari di Venezia Mestre-Venice Italy riccardo.marin@uam.es; b Nanomaterials for Bioimaging Group (nanoBIG), Departamento de Física de Materiales, Universidad Autónoma de Madrid Madrid Spain; c Institute for Advanced Research on Chemical Sciences (IAdChem), Universidad Autónoma de Madrid Madrid Spain; d CINBIO, Universidade de Vigo 36310 Vigo Spain; e Departamento de Física Aplicada, Universidade de Vigo 36310 Vigo Spain; f Transmission Electron Microscopy Characterization Laboratory, Department of Molecular Sciences and Nanosistems, Ca’ Foscari University of Venice Venice Italy

## Abstract

Copper-based semiconductor nanocrystals (SNCs) are a broad class of nanomaterials that include pnictogenides, chalcogenides, and halides. The breadth of compositions, crystal structures, and electronic properties displayed by these SNCs is reflected in their rich photophysics, which can underpin strong photon absorption capabilities, efficient (photo)luminescence, and plasmonic behavior. Because of this optical versatility, copper-based SNCs have been proposed for applications in fields such as photovoltaics, sensing, bioimaging, and photocatalysis, in some cases reaching the level of commercialization. In this review, we provide an up-to-date overview of the research on these nanomaterials with optical properties of outmost technological relevance. After a brief introduction on light-(nano)matter interaction, we individually discuss pnictogenides, chalcogenides, and halides, pinpointing structure–property relationships, identifying the most common synthesis approaches, and highlighting cases of application we consider particularly relevant or novel. Lastly, we outline outstanding challenges, with the hope of spurring the ingenuity and curiosity of researchers towards the next discoveries in this multicoloured and multifaceted field.

## Abstract 2

Copper-based semiconductor nanocrystals are versatile nanomaterials with diverse compositions, structures, morphologies, and optical properties, enabling applications in biomedicine, photocatalysis, photovoltaics, and lighting.

## Graphical abstract

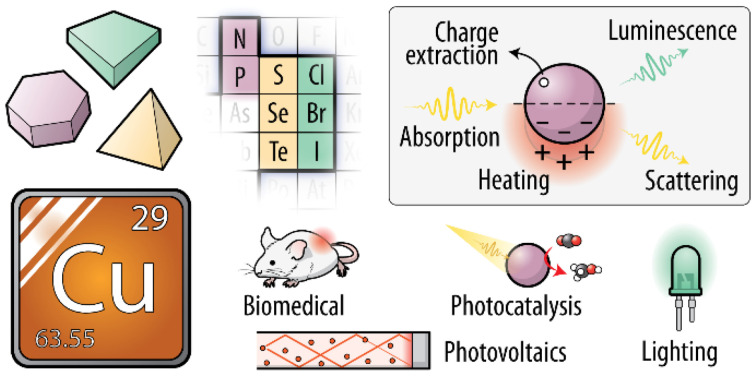

## 1. Introduction

Copper (Cu) is the 29^th^ element of the periodic table and has key technological relevance. Classified as a transition metal, it gets its name from the island of Cyprus, where it was copiously mined in ancient times. Copper as a metal has played a key role in the development of early civilizations, so much so that an almost 2000-year long period of human history (from approximately mid-5^th^ to early 3^rd^ millennium BC) is referred to as “The Copper Age”.^1^ During that prehistoric period, tools and weapons were mainly made of this rather malleable metal with a melting point of 1085 °C and capable of retaining sharp edges despite repeated use. Copper continued to play a central role also in the next era, “The Bronze Age”, since bronze is a Cu–Sn alloy. Even when iron took over the military and industrial scenes, copper remained a valuable metal used to produce copperware, jewelry, and mirrors. Moreover, it started to become a valuable coinage metal. Fast forward to more recent history: in the 1820s, copper wires began being used in electrical wiring, *e.g.*, for telegraph lines and later for telephone lines.^2^ Copper also features antimicrobial activity. Indeed, copper is the first solid antimicrobial material registered at the U.S. Environmental Protection Agency.^3^

But it is not only in its metallic form that copper has been used by humanity over the course of history: Ionic and non-ionic copper compounds have also found their place in human technology. CuSO_4_ was already used already by ancient Egyptians, Greeks, and Romans for medicinal purposes, in inks, and as a mordant in dyeing. Records of these uses also remain also in the Middle Ages, and in the 19^th^ century, it started to be used in electroplating, agriculture,^4^ and pest control. Cu-acetate has found similar application scenarios, while Cu(NO_3_)_2_ has been employed for textile dyeing, firework production, and in laboratory settings as an oxidizing agent and catalyst for organic reactions.^5^ CuCN is instead mainly used since the 18^th^ century in electroplating, and CuCl_2_ is a key catalyst in organic reactions.^1^

While the story and use of metallic copper and copper compounds spans several centuries and millennia, it is only in the 20^th^ century that Cu-based semiconductor materials began to be studied and applied. Indeed, Cu_2_O is one of the earliest-known semiconductor materials,^6^ with the first patent on its use as current rectifier being filed in 1925. Cu_2_S (chalcocite) and CuS (covellite) have been studied in the 1930s–1950s for their photoelectric properties. Since the 1970s, CuInSe_2_ has been heavily investigated for photovoltaic applications, becoming a central material in so-called CIGS solar cells.

Moving to the nanoscale – even though Cu-doped CdS and ZnS colloidal nanocrystals have been reported since the mid-1980s^7^ − the history of Cu-based semiconductor nanocrystals (SNCs) starts in the late 1990s, early 2000s. These nanomaterials feature intriguing optical properties that somehow remained elusive until recently. To that end, a first example of photoluminescent CuInSe_2_ nanocrystals was reported by Revaprasadu and co-workers,^8^ while the first report by the Burda group on the plasmonic properties of CuS nanocrystals was published in 2009.^9^ Since then, a plethora of studies on different copper-based SNCs have been published, reaching a tremendous level of understanding and control over the properties of these systems. The interest around Cu-based SNCs arises from the control that can be exerted on their optical properties through tuning of the composition, stoichiometry, structure, and copper's oxidation states.

Even though research on some of these nanomaterials peaked a few years ago, there is still a steady stream of publications on their fundamental properties and uses across a variety of fields, including biomedicine, energy conversion, catalysis, and lighting. Moreover, the recent push in the research on perovskite nanocrystals has led to the preparation of several Cu-based halide nanomaterials with unique properties.

**Fig. 1:**
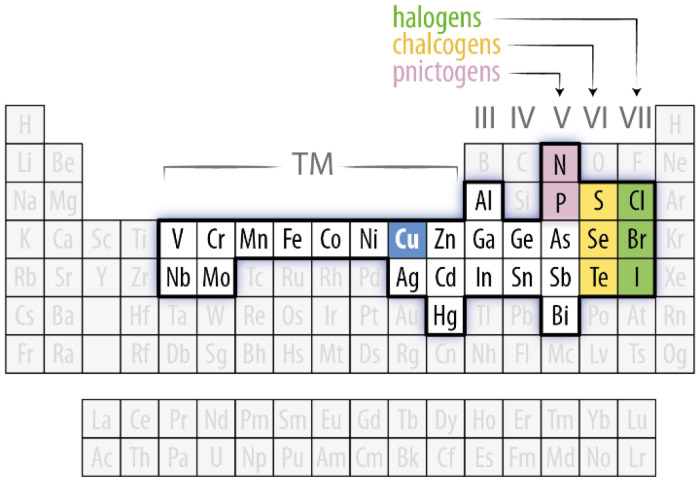
Overview of the elements of interest in the context of copper-based SNCs. Cu is highlighted in blue, alongside the elements belonging to the pnictogen (pink), chalcogen (yellow), and halogen (green) groups that define the families of SNCs discussed in this review. The other elements in white background are found in multinary and/or alloyed copper-based SNCs.

The persistent interest in Cu-based SNCs is highlighted by excellent reviews published on the subject over the past few years. These reviews have varying degrees of comprehensiveness: some have a focus on chalcogenides,^10–12^ others on halides,^13,14^ while others even narrow the discussion down to a specific subclass (*e.g.*, CuInS_2_ QDs^15^). Some reviews describe selected application scenarios^16,17^ and others – while remaining cornerstone pieces – have been published more than five years ago.^18^ This review proposes a fresh and comprehensive look at a wide span of nanomaterial compositions, yet it is circumscribed to the analysis of the optical properties and the proposed applications based on them (Table 1). Herein, we highlight the structure–property relationships for each Cu-based nanomaterial, also identifying similarities between different nanomaterial families. To accomplish this goal, we first introduce basic concepts of light–matter interaction in nanomaterials (section 2). We then move to sections identified by the nature of the anion in the nanomaterial composition (Fig. 1): pnictogenides (section 3), chalcogenides (section 4), and all-inorganic halides (section 5). Each section is further divided into subsections where selected families of Cu-based semiconductors are reviewed, touching upon their crystal structure, optical properties, synthesis methods, and applications. Whenever possible and relevant, parallels are drawn between different nanomaterial families. Lastly, we put the state-of-the-art of Cu-based nanomaterials in a broader context, highlighting future challenges and opportunities in this field (section 6).

**Table 1:** Main semiconductors discussed in this Review, their respective crystal structure, bulk bandgap (when possible, the direct or indirect nature of the bandgap is reported), optical properties as SNCs, and main application fields

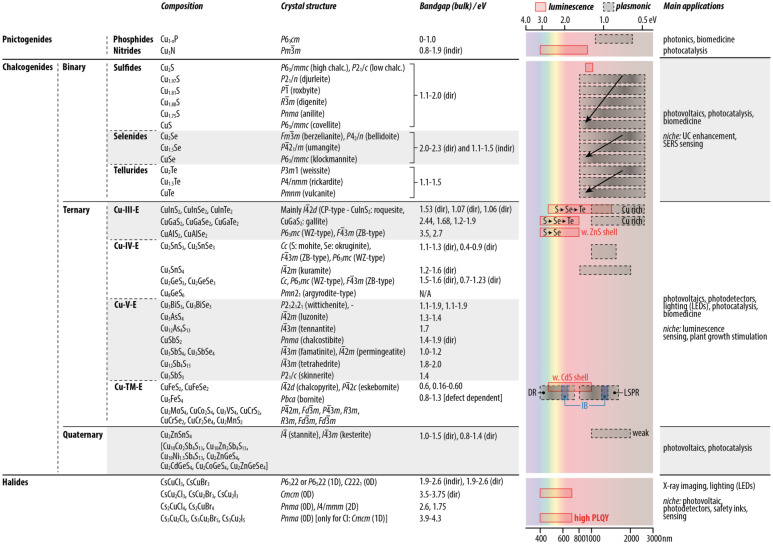

## 2. Brief phenomenological background on light–matter interaction at the nanoscale

**Fig. 2:**
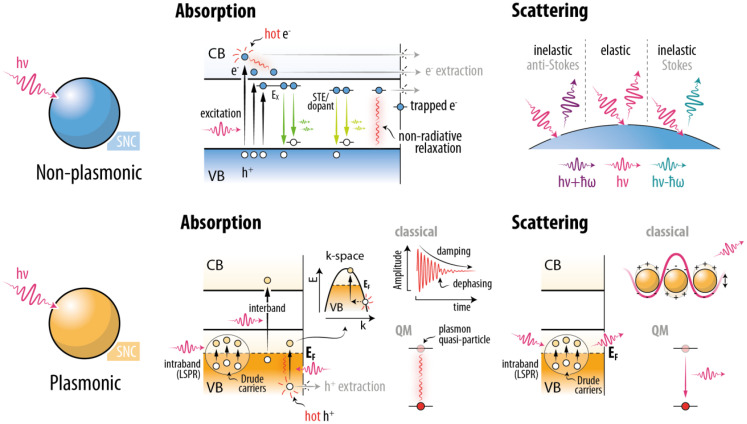
Summary of the modalities of light–matter interaction in non-plasmonic and plasmonic SNCs.

Before delving into Cu-based nanocrystals, it is useful to recall some basic concepts of light–matter interaction that guide the discussion on the optical properties of the nanomaterials presented in this Review (Fig. 2). Broadly speaking, the means of interaction of an optical nanomaterial with photons are two-fold: absorption and scattering. The specifics of the underlying physical mechanisms vary depending on whether the nanomaterial displays plasmonic properties or not. Thus, we divide the discussion into plasmonic and non-plasmonic semiconductor nanomaterials. Moreover, we focus on the interaction with UV-Vis-NIR photons – *i.e.*, from approximately 250 to 2500 nm. We should also highlight that we do not aim for a rigorous mathematical description of the phenomena.

### 2.1. Non-plasmonic materials

#### 2.1.1. Absorption

In non-plasmonic materials, absorption events occur when the energy of the impinging photon matches the energy gap between two real electronic/energy states: One occupied and the other unoccupied. In SNCs, these states generally belong to the conduction and valence bands (CB and VB) or are introduced by dopants. Photoexcitation generally leads to the formation of free excitons (E_x_): electron–hole pairs whose energy is stabilized *via* Coulomb interaction to a value (optical bandgap) lower than the energy difference between the CB bottom and the VB top (electronic bandgap). When the excited electron is promoted to an energy state above the lowest CB energy state, it is referred to as “hot electron”, since it has excess energy compared to thermal equilibrium. A hot electron can subsequently get trapped at a defect state related to vacancies, dangling bonds, or surface ligands. Alternatively, it can reach the bottom of the CB *via* thermalization, *i.e.*, transfer of momentum to the crystal lattice in the form of lattice vibrations (phonons) to reach the band edge.^15^ This process constitutes the generation of heat. From the bottom of the CB, the electron can occupy a free exciton state or be further stabilized in localized states due to, *e.g.*, electron–phonon interaction – self-trapped exciton (STE) – or presence of dopant-related states. From one of these states, the electron can radiatively recombine with a delocalized or localized (*i.e.*, occupying an energy state just above the VB) hole: A process referred to as photoluminescence. Note that even in the case of free-exciton luminescence, there is an energy difference between the absorption and emission photon (Stokes shift). The origin of this shift – whose explanation is beyond the scope of this review – include the presence of dark and bright exciton states (which are respectively inactive and active in optical transitions), energy transfer among different-sized nanocrystals, and electron–phonon coupling.^16^ Alternatively, the radiative transition can occur with the involvement of interbandgap states, *e.g.*, introduced by dopants. Excited electrons (in exciton or trap states) can also undergo non-radiative relaxation processes. Lastly, hot electrons, electrons at excitonic states, and in trap states can all be extracted from the semiconductor and transferred to another species, generally molecules. This process is harnessed in photocatalysis. Note that similar considerations made for hot electrons can be made for hot holes, with the caveat that the processes take place in the VB instead (see Fig. 2).

#### 2.1.2. Scattering

When photons impinge on a nanomaterial, their electromagnetic field can be perturbed by the encounter with a system having a refractive index different from the one of the medium in which the photons are propagating. The photons are therefore scattered. Most photon scattering events are elastic in nature, meaning that the energy of the photon is conserved in the process. Within the elastic regime, scattering can be described more accurately using one of two formalisms depending on the ratio between the characteristic nanomaterial size (*r*) and the wavelength of the impinging photon (*λ*): Mie (*r*/*λ* ≳ 1) and Rayleigh (*r*/*λ* ≪ 1) scattering. Mie scattering is characterized by preferential forward scattering and geometry-dependent resonance modes, while Rayleigh scattering is more isotropic and scales with the inverse fourth power of the wavelength of incident photons.

Inelastic scattering events involve energy exchange with the crystal structure and, thus, are non-conservative in nature. The resulting photons have either lower or higher energy than the incident one: these events are referred to as Stokes and anti-Stokes scattering, respectively. Raman spectroscopy analyses these photons to obtain information about the material under study.

### 2.2. Plasmonic materials

In plasmonic nanomaterials, a complete description of the light–matter interaction should consider both optical electronic transitions (see above) and the activation of confined, resonant, collective charge carrier oscillations referred to as localized surface plasmons resonance (LSPRs). The charge carriers participating in these oscillations are either quasi-free electrons or quasi-free holes close to the Fermi state, which falls within an electronic band rather than within the bandgap. From a classical perspective, the external oscillating field drives currents within the nanostructure, constituted by charge carriers in a partially filled band. The mobility and density of these carriers, together with the geometry of the structure, determine the natural oscillation frequencies that can be excited resonantly. This description is consistent with the framework of the Drude model and can be seen as the collective excitation of carriers to states near the Fermi level through intraband transitions. These collective excitation events constitute the excited currents and the LSPR. An alternative perspective, treating both light and the collective response in the material within a quantum mechanical framework, describes the plasmon as a quasiparticle (a boson) that represents a quantized, collective oscillation mode. A photon can therefore interact with a plasmonic nanomaterial when its energy matches the energy of the plasmon (quantum interpretation), which is directly related to the collective charge carrier oscillation frequency (classical interpretation).

Because of the topic of this review, it is useful to briefly highlight the unique features of plasmonic SNCs. Note that an in-depth discussion on this topic was published by Faucheaux *et al.*,^19^ and the interested reader is redirected to that publication. While in metal nanoparticles the main charge carriers supporting the LSPR are electrons (negative charges), in SNCs holes (positive charges) in the VB are the main responsible for the plasmonic behavior. In other terms, as the conductive phenomenon occurs close to the high-energy edge of the VB, the band curvature and population – which gives rise to the properties of the hole – also determine the conductivity and plasmonic properties of the system. A key differentiator between metal nanocrystals and SNCs is the charge density (*N*), which is orders of magnitude lower in SNCs (10^16^–10^21^ cm^−1^) compared to their metal counterpart (approx. 10^23^ cm^−1^).^19^ The bulk plasma frequency of a material scales with the charge carrier density according to:1
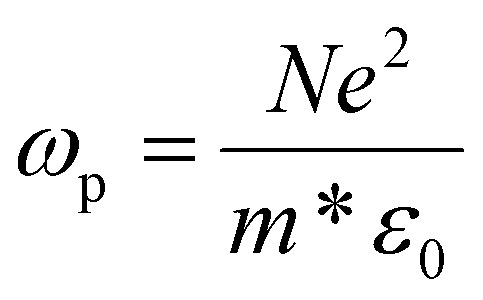
where *e* is the elementary charge, m* is the charge carrier effective mass, and *ε*_0_ is the vacuum permittivity.

Thus, a lower charge density translates to a lower plasma frequency (energy), which corresponds to longer wavelengths. Indeed, plasmonic SNCs show LSPR modes centered in the NIR-midinfrared (MIR) range, while metallic plasmonic nanocrystals are mainly active in the UV-visible. This extension of the range where plasmonic materials can operate using SNCs comes at the cost of an overall lower photon extinction cross section (*vide infra*). Importantly, in SNCs the carrier density can be tuned and dynamically controlled by intentional – and often reversible – introduction of vacancies, as well as by redox or electrochemical means. This charge density tuning offers an additional “knob” to modulate the optical properties of SNCs compared to metallic nanocrystals, where LSPR energy can be mainly modulated through size, morphology, and composition.

#### 2.2.1. Absorption

Photon absorption events in plasmonic nanomaterials can occur due to the activation of LSPRs (supported by intraband electronic transitions) or can prompt interband electronic transitions. In the former case, the induced charge carrier oscillation undergoes a damping/dephasing. In the quantum mechanical interpretation, this corresponds to the plasmon quasiparticle decaying. In so doing, the energy can be dissipated through scattering events between the charge carriers and phonons or defects. This “frictional” decay occurs for excited charge carriers (*i.e.*, quasi-free carriers in the band of the majority carriers) interacting with the nuclei in the material structure, and it leads to heat generation by transferring their kinetic energy to phononic modes. More rarely, the energy of the decaying plasmon can be transferred to charge carriers close to the nanoparticle border, where scattering events can support the generation of hot carriers.^20^ Hot carriers are also generated by direct optical interband excitation events. Like in the case of non-plasmonic materials, these hot carriers can either thermalize – thus generating heat – or be transferred to external moieties (*e.g.*, molecules).

#### 2.2.2. Scattering

When the activated LSPR mode decays radiatively, the nanoparticle is said to have scattered the photon exciting it. This is an elastic scattering event in that the re-emitted photon has the same frequency as the exciting one. Scattering is dominant in larger plasmonic nanoparticles, scaling in the quasistatic approximation as the square of their polarizability rather than linearly like the absorption; but they also depend on their aspect ratio and overall geometry.^21^ Plasmonic nanoparticles with large scattering cross sections function as effective nanoantennas operating at visible and IR wavelengths, with their excitation dipoles coupling strongly with the far field.

### 2.3. Some important metrics

#### 2.3.1. Extinction

The combined probability of photon scattering and absorption is referred to as photon extinction. The scattering, absorption, and extinction efficiency of a system are expressed by the respective cross sections (*σ*_sc_, *σ*_abs_, *σ*_ext_). Molar absorption and extinction coefficients are also used (*ε*_abs_, *ε*_ext_). One should note that in single-crystal optical materials (the background field of many researchers also working on optical nanomaterials), extinction and absorption are often used interchangeably; and rightfully so, since scattering is generally negligible in those systems. However, in nanomaterials, sometimes the scattering and absorption cross sections can be of similar order of magnitude, or scattering can even dominate. This is true, for example, in larger plasmonic nanomaterials or when nanoparticles aggregate to yield larger individual units. Careful distinction of scattering and absorption is therefore pivotal in the study of the optical properties of nanomaterials. For example, depending on the application, one would want to maximize solely the absorption or the scattering (rather than the total extinction!) of a nanomaterial.

#### 2.3.2. Heat generation

Non-radiative recombination events lead to localized heat. The figure of merit for this photon-to-heat process is the heat conversion efficiency (HCE), which is defined as the fraction of energy converted to heat over the total amount of energy extinguished by a system.^22,23^2
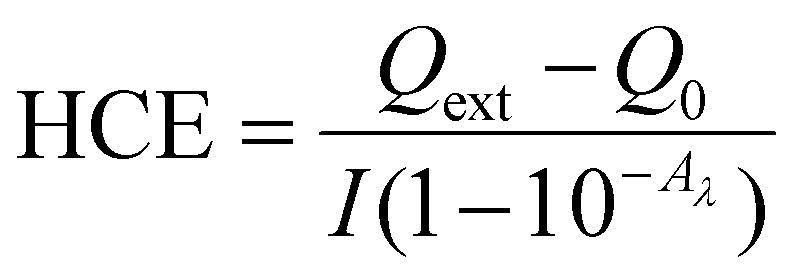
here *Q*_ext_ is the external heat flux, *Q*_0_ is the heat generated by the dispersant (in the case of a SNC dispersion), *I* is the laser power, and *A*_*λ*_ is the absorbance at the laser wavelength.

The “external” version of this figure of merit (eHCE) has also been proposed, which considers also the mass of photon absorbing material.^24^3
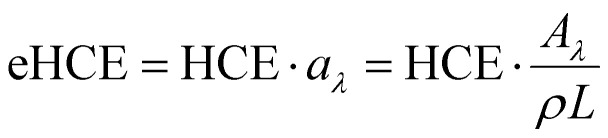
where *a*_*λ*_ is the mass absorption coefficient of the species under study at the excitation wavelength, *ρ* is the mass concentration (in mg mL^−1^) and *L* is the optical path (in cm) of the setup used to experimentally measure these metrics.

#### 2.3.3. Photoluminescence

Photoluminescence quantum yield (PLQY; the ratio between emitted and absorbed photons) is widely regarded as the key figure of merit for photoluminescence.4
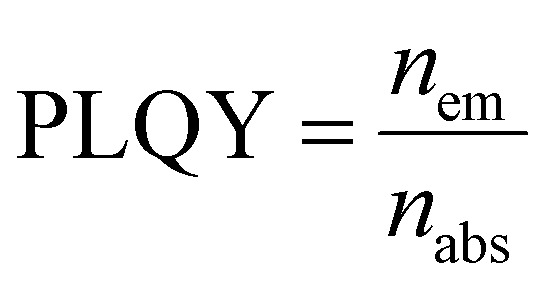


However, brightness (obtained as the product of PLQY and absorption cross section) is a more complete metric that considers both photon absorption and emission efficiency.^25^

5 *B* = *σ*_abs_·PLQY

#### 2.3.4. Photocatalysis

Alongside creating photogenerated currents in photodetection devices, a key application of extracted electrons is in photocatalysis/photochemistry. While there are several metrics for quantifying the efficiency of photocatalytic processes, internal and external quantum efficiencies are the most used one. The former considers the number of absorbed photons, while the latter the total number of excitation photons. The extern quantum yield, also referred to as apparent quantum efficiency (AQE), is therefore defined as the ratio between reacted electrons and absorbed photons, or – equivalently – between reaction rate (*R*) and photon flux (*ϕ*).^26^6
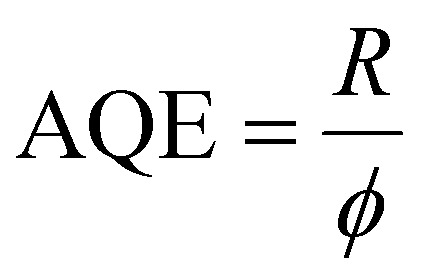


#### 2.3.5. Quantum confinement

An important parameter in the context of SNCs is the exciton Bohr radius.^27^ This magnitude (*a*_B_) is the characteristic size of the quasiparticle exciton (an electron–hole pair interacting through Coulomb interactions), which is a function of the effective mass of the electrons and holes in a specific material.7
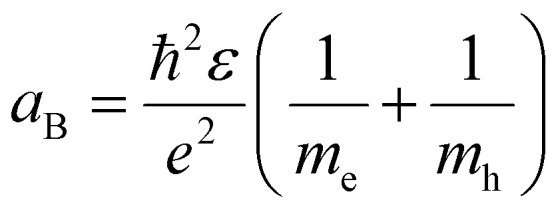
where *ħ* is the reduced Planck's constant, *ε* is the permittivity of the material, *m*_e_ and *m*_h_ are the electron and hole masses. When the size of the SNC approaches and gets smaller than the exciton Bohr diameter (2*a*_B_), the SNC is said to enter the quantum confinement regime. In this regime, the energy difference between VB and CB increases (*i.e.*, the bandgap widens) following the particle-in-a-box model – hence the absorption and, if present, emission properties of the SNC can be controlled by changing the SNC size. In this Review, we decided to always use the more general term SNCs rather than the possibly more common and now mainstream term of quantum dots (QDs). That is because, strictly speaking, QDs are only those SNCs in quantum confinement regime. SNCs is thus a more generally applicable term.

After this introduction on the basics of light-nanomatter interaction, we focus our attention on the different families of Cu-based SNCs.

## 3. Pnictogenides

### 3.1. Copper phosphides

#### 3.1.1. General considerations, structure, and optical properties

**Fig. 3:**
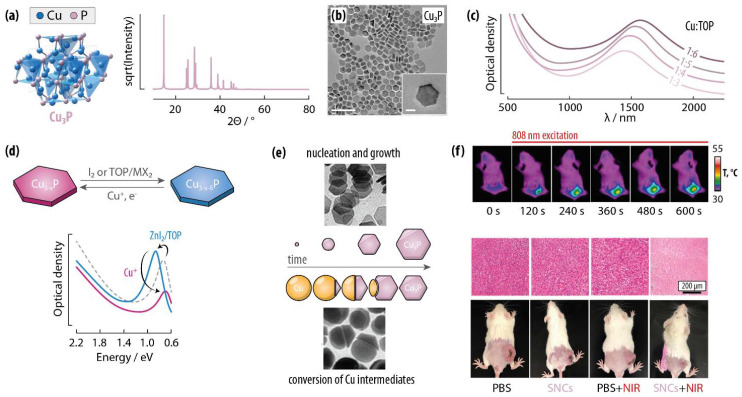
Copper phosphide SNCs. (a) Unit cell and simulated X-ray powder diffraction (XRPD) pattern of the Cu_3_P phase generally reported in SNCs. (b) Transmission electron microscopy (TEM) image of Cu_3_P SNCs showing the characteristic hexagonal nanoplate morphology. Adapted with permission from ref. 29, Copyright 2016, John Wiley and Sons. (c) Extinction spectra of Cu_3_P SNCs synthesized with different Cu : TOP feeding ratios. The LSPR peak shifts at longer wavelengths with increasing TOP content. Adapted with permission from ref. 29, Copyright 2016, John Wiley and Sons. (d) Scheme outlining the reversible redox process that allows controlling the carrier density and hence the LSPR peak position and intensity in Cu_3−*x*_P SNCs. Adapted from ref. 37, Copyright 2024, American Chemical Society. (e) Two possible Cu_3_P SNC growth mechanisms: homogeneous nucleation and growth (top) or gradual phosphorization of metallic Cu nanoparticles (bottom). Adapted with permission from ref. 30, Copyright 2012, American Chemical Society. (f) An example of Cu_3_P SNC use in nanomedicine: thermal images of a tumour model during Cu_3_P SNC-mediated photothermal therapy (top) and comparison of the effect in control models (bottom). The reported H&E-stained tumour sections and the photos of mice at the end of the treatment show a marked tumour reduction. Adapted with permission from ref. 38, Copyright 2021, American Chemical Society.

Together with copper chalcogenides (Cu–E; see section 4.1), copper phosphide (Cu_3−*x*_P) nanocrystals belong to the family of self-doped doped SNCs featuring plasmonic properties. As the generic formula used in this review suggests, Cu_3−*x*_P nanocrystals can be prepared in a variety of compositions with varying relative copper content. A theoretical study by Harper *et al.* identified several compositions (CuP_10_, Cu_2_P_7_, CuP_2_, Cu_2_P, and Cu_3_P) each with a unique crystal phase.^28^ The nanocrystals reported in the literature crystallize in the *P*6_3_*cm* Cu_3_P hexagonal phase (Fig. 3a).^29,30^ Correspondingly, the SNCs have a hexagonal nanoplate morphology (Fig. 3b).^31^ Cu_3−*x*_P in the *P*6_3_*cm* phase has been recently suggested to be a semimetal^32^−and hence it does not display a bandgap in the classical sense. Yet some studies show well-defined bandgaps with varying values up to >1.0 eV.^33,34^ Cu_3−*x*_P SNCs are generally sub-stoichiometric in copper content, featuring free – here synonym of unbound-charge carriers (holes) in the VB and hence a p-type behavior. This characteristic stems from the fact that the introduction of copper vacancies is thermodynamically favorable. The presence of copper vacancies in the structure also facilitates ion diffusion, thus enabling cation exchange processes.^35^ In fact, Cu_3−*x*_P nanocrystals have been used as sacrificial species for the preparation of, *e.g.*, InP nanocrystals.^36^

As far as the optical properties are concerned, the positive charge carriers support LSPR modes centered in the NIR-to-MIR range (Fig. 3c).^29^ The position of the LSPR peaks can be tuned by modulating the charge carrier density (*N*_H_) in the nanocrystal. This strategy has been leveraged by Rachkov *et al.*, who developed Cu-coupled reversible redox strategies to controllably modify *N*_H_ – which lies in the 10^21^–10^22^ cm^−3^ range for Cu_3−*x*_P nanocrystals.^35^ Specifically, Cu_3−*x*_P → Cu_3−*x-δ*_P oxidation was promoted in the presence of I_2_ or metal halides and TOP. This process could be reversed by exposing the nanocrystals to Cu^+^ in the form of tetrakis(acetonitrile)copper(i) hexafluorophosphate ([Cu(MeCN)_4_]PF_6_). The position of the LSPR peak center could be tuned with this strategy by 200 meV in the 700–900 meV range (Fig. 3d). It should be noted that this controlled redox approach draws inspiration from the protocols developed for copper chalcogenide nanocrystals, which have an analogous plasmonic behavior (see below).

#### 3.1.2. Synthesis

In one of the first reports on this nanomaterial, Manna *et al.* prepared Cu_3−*x*_P SNCs by bubbling PH_3_ as phosphorous source – obtained *ex situ* from the reaction between Ca_3_P_2_ and HCl – into a solution of CuCl in a mixture of trioctyl phosphine (TOP), trioctyl phosphine oxide (TOPO), and oleylamine (OAm).^34^ Therein, TOP acted as SNC size tuning agent by determining the nucleation rate and hence the total number of prepared nanocrystals. The central role of TOP in the preparation of Cu_3−*x*_P SNCs was also highlighted in a report by De Trizio *et al.*^30^ The authors developed a one-pot protocol whereby a CuCl solution in an alkylamine mixture is injected into a TOP and TOPO mixture at high temperature (370 °C). Depending on the amount of TOP in the reaction mixture, a final nanoplate morphology was obtained *via* homogeneous Cu_3−*x*_P nucleation and growth or a gradual phosphorization of Cu intermediate nanocrystals (Fig. 3e). In the latter case, the conversion proceeded through growth along two main epitaxial Cu/Cu_3−*x*_P interfaces to yield Janus Cu–Cu_3−*x*_P nanocrystals that ultimately produced Cu_3−*x*_P nanoplates (Fig. 3c).^30^ Liu *et al.* proposed the preparation of Cu_3−*x*_P SNCs *via* hot injection of a tris(trimethylsilyl)phosphine (TMS)_3_P solution in 1-octadecene (ODE) into a CuCl solution in TOP/OAm kept at up to 180 °C. Intriguingly, a “wet annealing” process carried out at 300 °C led to a reshaping of the hexagonal nanoplates into disks, likely owing to the OAm-driven etching.^29^ More recently, Rachkov *et al.* reported on a one-pot synthesis approach using copper halides, tris(diethylamino)phosphine [P(NEt_2_)_3_], OAm, and trioctylamine.^39^ Therein, the authors placed the nucleation event at approximately 230 °C and identified the P/Cu ratio in the reaction mixture as the key parameter to control the nanocrystal size. Thorough investigation of the reaction intermediates *via* nuclear magnetic resonance (NMR) shed light on the nature of the Cu–P species involved in the nanocrystal nucleation and growth. The synthesis of these SNCs was also reported *via* single-source precursor [Cu(H)(PPh_3_)]_6_ by Downes *et al.*, who also identified the presence of a Cu nanocrystal intermediate gradually converted to Cu_3−*x*_P.^40^

#### 3.1.3. Applications

Cu_3−*x*_P SNCs have been proposed in fields including electrocatalysis, photonics, and nanomedicine. For example, Mondal *et al.* used Fe-doped Cu_3_P SNCs for water splitting, while Downes *et al.* showcased the potential of these nanomaterials for CO_2_ reduction.^41^ Liu *et al.* proposed instead the use of Cu_3_P SNCs for laser applications in the telecom band. The carefully-tuned LSPR band at 1.5 μm made them suitable as nonlinear absorbers in a high-energy Q-switched laser.^29^ Cu_3_P SNCs have also been shown to be effective photothermal (PT) agents and photosensitizers (PS) in photothermal and photodynamic therapy, respectively (Fig. 3f).^31,38^ The LSPR properties of Cu_3_P SNCs were also harnessed by Sun *et al.* to enhance the performance of graphene-based photodetectors, identifying the nature of the capping ligands as a key parameter in enabling good performance.^40^

### 3.2. Copper nitrides

#### 3.2.1. General considerations, structure, and optical properties

**Fig. 4:**
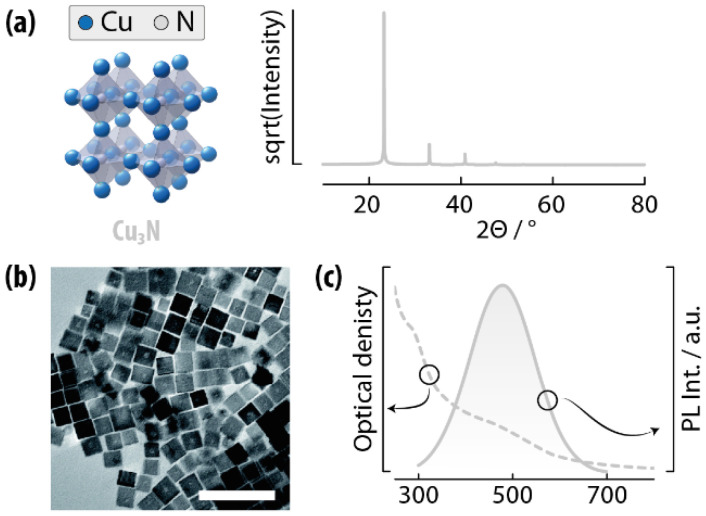
Copper nitride SNCS. (a) Unit cell and simulated XRPD pattern of Cu_3_N. (b) TEM image of Cu_3_N SNCs showing the characteristic cubic morphology. Scale bar is 100 nm. (c) Extinction and emission spectrum of Cu_3_N SNCs. Adapted with permission from ref. 44, Copyright 2020, Royal Society of Chemistry.

Cu_3_N crystallizes in a cubic anti-ReO_3_ structure (space group *Pm*3̄*m*), where the Cu atoms occupy the corners and face centers of the cube, and nitrogen atoms sit at the body center, forming a 3D open framework (Fig. 4a).^42^ The morphology of the SNCs in this phase is also cubic (Fig. 4b). Moreover, the crystal structure is amenable to copper replacement with other metal ions. In fact, similarly to other Cu-based nanomaterials (see below), Cu_3_N SNCs can act as cation exchange platforms for the synthesis of SNCs, such as Ni_4_N and CoN.^43^

In its bulk form, copper nitride (Cu_3_N), is a semiconductor with a calculated indirect bandgap of 1.0 eV, while experimental reports show values in the 0.8–1.9 eV range – depending on the preparation method and actual stoichiometry. Aside from high values of bulk absorption coefficient (10^4^–10^5^ cm^−1^),^45–47^ Cu_3_N in the form of SNCs has been shown to exhibit electro- and photoluminescence in the visible-NIR region (Fig. 4c).^44,48,49^

#### 3.2.2. Synthesis

The synthesis of Cu_3_N SNCs typically involves the reaction of copper salts with nitrogen precursors under reducing or inert conditions. A common approach proceeds through the thermal decomposition of a copper compound in the presence of an organic amine, which serves as both solvent and nitrogen source. The reaction is typically carried out at 220–260 °C under inert atmosphere, producing colloidally stable nanocubes or quasi-spherical nanocrystals. Explored copper precursors are Cu(NO_3_)_2_, copper methoxide (Cu(OMe)_2_), and copper acetate. Typically employed amines are OAm, octadecylamine, and benzylamine. Alternatively, urea and ammonia were also explored as nitrogen sources, conducting the reaction in high-boiling point alcohols such as 1-nonanol and decreasing the length of the aliphatic chain all the way down to 1-pentanol.^48^ The formation mechanism of Cu_3_N SNCs entails an initial nucleation of small, quasi-spherical nanoparticles, followed by their ripening into nanocubes. Prolonging the reaction time beyond the formation of the nanocubes led to SNC degradation, with the appearance of metallic copper. A recent study by Zheng *et al.* showed the possibility to grow Cu_3_N nanorods using Cu-acetate and OAm in a heat up approach, given the preferential adsorption of the ligands on the SNC so to favor growth along the [100] direction.^50^ The nature of the amine was critical in the morphology and quality of the final crystals, albeit the authors did not provide a definitive explanation for this effect. The presence of O_2_ in the reaction environment was shown to be pivotal in obtaining homogeneous morphology and size, hence the authors suggested an active role of this molecule as oxidizer of aminated species during the SNC synthesis.

Among the publications on the subject, the manuscript by the group of De Roo^51^ stands out as a pleasant example of a didactical discussion on the key role that the purification step plays in the preparation of high-quality, stable colloidal SNCs.

#### 3.2.3. Applications

Reported examples of uses of Cu_3_N SNCs involving light include mainly their use in photocatalysis.^52,53^ The most relevant is the report by Barman *et al.*, who showed that coupling of these SNCs with Au nanoparticles affords photocatalysts for organic dye degradation, thanks to the favorable band alignment between Au and the semiconductor.^54^ Dasgupta *et al.* recently reported on Cu_3_N-Ag SNCs for preparing a nanostructured electrode to be used in food spoilage detection systems.^55^ The NH_3_ sensor works through a chemiresistive working principle, which is enhanced under UV exposure. This effect stems from the presence of Cu_3_N-Ag SNCs, which promote electron–hole separation. Cu_3_N SNCs have also been suggested as candidates for absorbing materials in photovoltaic devices, but we could not find reports on this use.

## 4. Chalcogenides

### 4.1. Binary compounds

#### 4.1.1. General considerations, structure, and optical properties

Binary copper chalcogenides (Cu_2−*x*_E, E = S, Se, Te) SNCs are the prototypical example of plasmonic semiconductor nanomaterials, with the first report on Cu_2−*x*_S SNCs by Luther *et al.* in 2011.^56^ Cu_2−*x*_E SNCs are self-doped p-type semiconductors, with copper vacancies introducing free/unbound holes that result in tunable localized surface plasmon resonance (LSPR) in the NIR-to-MIR range. A special case is represented by CuS (covellite) SNCs, where the delocalized holes in the VB do not arise from copper vacancies (whose formation is energetically unfavored), but rather from the intrinsic band structure of the nanomaterial.^57^ Carrier densities reaching ∼10^21^–10^22^ cm^−3^ were reported – a value that approaches metallic regimes.

##### Copper sulfides

**Fig. 5:**
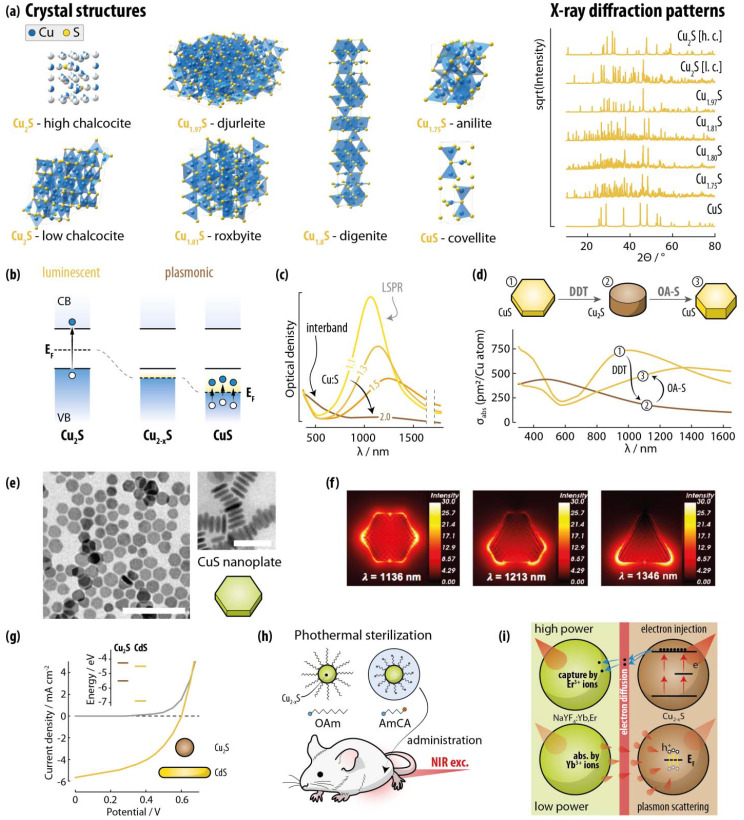
Copper sulphide SNCs as representatives of the binary copper chalcogenide family. (a) Unit cell and simulated XRPD patterns of the most reported Cu_2−*x*_S compositions. (b) While Cu_2_S displays the electron structure of a typical SNC (with the Fermi level in the bandgap), Cu_2−*x*_S compositions with *x* > 0 have excess holes in the VB and hence the Fermi level falls within this band. As such, LSPR-supporting intraband transitions can take place in SNCs with these compositions. (c) Extinction spectrum of Cu_2−*x*_S SNCs with different Cu : S values, showing a gradual decrease in the LSPR mode intensity with increasing Cu^+^ content. Adapted with permission from ref. 58, Copyright 2013, American Chemical Society. (d) Reversible transformation between CuS and Cu_2_S SNCs through the use of DDT and OA-S, alongside the corresponding change in the extinction spectra. Adapted with permission from ref. 61, Copyright 2017, American Chemical Society. (e) TEM images of CuS SNCs showing the typical nanoplate morphology of this class of SNCs. Scale bars are 100 (left) and 50 (right) nm. Reprinted with permission from ref. 57, Copyright 2013, American Chemical Society. (f) Near-field intensity maps calculated at the resonant condition for CuS SNCs with different morphologies for in-plane illumination. Reprinted with permission from ref. 59, Copyright 2019, American Chemical Society. (g) Current density–voltage characteristics of a photovoltaic device implementing Cu_2_S–CdS nanocrystals under no illumination (black) and under standard illumination (red). Adapted with permission from ref. 62, Copyright 2008, American Chemical Society. (h) Use of Cu_2−*x*_S SNCs as light-driven sterilants. ref. 63. (i) Two mechanisms of upconversion emission enhancement in NaYF_4_:Yb,Er supported by Cu_2−*x*_S SNCs. Electron injection to Er^3+^ ions occurs at high excitation powers, while plasmonic scattering dominates at low excitation powers. Adapted with permission from ref. 64, Copyright 2016, American Chemical Society.

Materials of the Cu_2−*x*_S family have direct bandgaps in the 1.1–2.0 eV range, depending on the actual composition of the material.^11^ Indeed, Cu_2−*x*_S (1 < *x* < 2) exhibits multiple crystalline forms, including chalcocite (Cu_2_S; high chalcocite, *P*6_3_/*mmc*; low chalcocite, *P*2_1_/*c*), djurleite (Cu_1.97_S; *P*2_1_/*n*), roxbyite (Cu_1.81_S; *P*1̄), digenite (Cu_1.8_S; *R*3̄*m*), anilite (Cu_1.75_S; *Pnma*), and covellite (CuS; *P*6_3_/*mmc*) (Fig. 5a). In all these materials, copper is found almost exclusively in its Cu^+^ oxidation state, while sulfur can take up a valency of −1 (CuS) or −2 (Cu_2_S).^58^ Note that all the above-mentioned structures have well-defined stoichiometries. Yet, some of them tolerate introduction of copper vacancies, which induces deviations from the theoretical compositions. Importantly, the more the stoichiometry deviates from Cu_2_S, the higher the value of *N*_H_. That is to say, the higher the value of *x* in Cu_2−*x*_S, the more charge carriers (holes) are present in the VB and, hence, the stronger the LSPR (Fig. 5b). The first report by Luther *et al.* showed a NIR LSPR peak for djurleite SNCs whose position corresponded to a hole density of 10^21^ cm^−3^ and, hence, a composition Cu_1.93_S. Yet, CuS supports the strongest LSPR modes (Fig. 5c and d). Its SNCs generally grow along preferential crystallographic directions 〈010〉, 〈100〉, and 〈11̄0〉, often yielding a triangular or, more often, hexagonal nanoplate morphology (Fig. 5e and f). The nanoplate morphology of these SNCs results in both in-plane and out-of-plane LSPR modes.^57,59^ Moreover, very recently *via* single-particle study using electron energy loss (EEL) spectroscopy, Elibol *et al.* showed that CuS SNCs in the form of thin nanoplates feature a plethora of plasmonic modes.^60^ The same authors also observed several visible and NIR emission signals under electron beam exposure.

Control over the plasmonic behavior in Cu_2−*x*_S – with LSPR peaks in the 0.4–1.5 eV range – can be achieved through redox reactions and/or cation removal/addition.^65^ To that end, exposure to air, I_2_, Br_2_, methyl viologen, or Ce^4+^ complexes oxidizes the SNCs,^66–68^ introducing excess holes. Along the same line, electrochemical redox of copper (Cu^+^ ↔ Cu^2+^) in CuS nanoplates allows to reversibly switch on–off the NIR LSPR mode.^69^ Liu *et al.* also showcased a reversible crystal phase interconversion between CuS and Cu_2_S enabled by exposure to 1-dodecanethiol (DDT) or OA and sulfur.^61^ This process proceeds through reshaping of CuS hexagonal nanoplates to Cu_2_S nanodisks, and back to nanoplates (Fig. 5d). These structural and morphological changes were also accompanied by corresponding variations in the optical properties. Similarly, the group of Houtepen demonstrated that CuS nanoplates deposited on an ITO substrate, when exposed to Cu^+^ ions, incorporate the metal ions thus converting irreversibly to Cu_2_S.^70^ This compositional and structural change is accompanied by the loss of plasmonic behavior and the appearance of NIR photoluminescence. This observation was consistent with the NIR emission (centered at 1.3 eV) recorded in Cu_2_S SNCs by the group of Alivisatos.^62^ Interband electronic transitions are also observed for Cu_2−*x*_S SNCs but are usually less discussed for these nanomaterials. The interband electronic transitions give rise to absorptions in the high-energy part of the spectrum (<500–600 nm), with quantum confinement effects pushing them further to the blue for sizes below 10 nm.^56^

##### Copper selenides

Cu_2−*x*_Se materials have been reported with a varying indirect bandgap of 1.1–1.5 eV and a direct bandgap in the 2.0–2.3 eV range. Cu_2−*x*_Se has a rich variety of crystal phases too.^71,72^ One of the most relevant composition is Cu_2_Se, which can crystallize in a cubic (*Fm*3̄*m*, berzelianite) and tetragonal (*P*4_2_/*n*, bellidoite) phase. An hexagonal, wurtzite-like, metastable phase has been observed too in SNCs.^73,74^ Other phases include umangite (Cu_1.5_Se; *P*4̄2_1_/*m*) and klockmannite (CuSe; *P*6_3_/*mmc*), alongside various non-stoichiometric compounds that crystallize in cubic, hexagonal, and trigonal structures. The optical properties and strategies to tune the properties of Cu_2−*x*_Se SNCs are similar to the ones outlined above for binary copper sulfides,^68,73,75^ with the value of *N*_H_ usually found in these SNCs being comparable to the ones in the Cu_2−*x*_S SNCs.^76^ Also similar is the amenability of these SNCs to be used as sacrificial nanomaterials in cation exchange reactions for producing other SNCs such as PbSe, HgSe, ZnSe, CdSe,^74,77^ and even ternary compounds including CuZnSe_2_ and CuSnSe_2_.^78^ As shown by the group of Manna, cation exchange reactions are boosted when non-stoichiometric, Cu vacancy-containing Cu_2−*x*_Se SNCs are used instead of fully stoichiometric Cu_2_Se SNCs.

##### Copper tellurides

Cu_2−*x*_Te show bandgaps in the 1.1–1.5 eV range. Though less explored, Cu_2−*x*_Te SNCs also support LSPR modes.^79,80^ Reported compositions include weissite (Cu_2_Te, *P*3*m*1), rickardite (Cu_1.5_Te, *P*4/*nmm*), vulcanite (CuTe, *Pmnm*), and other non-stoichiometric phases that crystallize in hexagonal, orthorhombic, and cubic structures.^11^ These SNCs also display LSPR activity supported by delocalized holes (especially the sub-stoichiometric compositions)^80,81^ and they can be used as sacrificial SNCs in cation exchange reactions.^82^

Note that alloys of different Cu_2−*x*_E can also be prepared.^83^ This alloying (*i.e.*, compositional control) is in fact a means to tune the plasmonic properties of the final SNCs. It should also be noted that the fewer reports available on selenides and tellurides are the result of fewer anionic precursors available for these chalcogens.^11^

#### 4.1.2. Synthesis

**Fig. 6:**
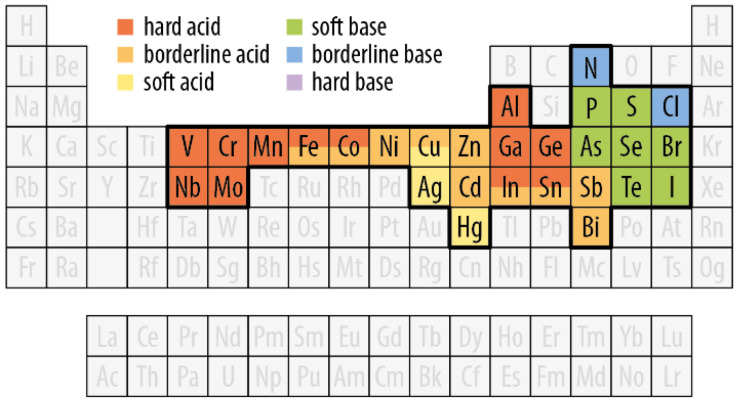
Lewis acid/base nature of the elements considered in this Review.

Over twenty years of research on Cu_2−*x*_E SNCs, several synthesis approaches have been developed for these nanomaterials, achieving control over size, morphology, crystal phase, composition, surface chemistry, and hence optical properties. Generally, the synthesis entails the use of copper and chalcogen sources (at times single-source precursors, *i.e.*, copper complexes with chalcogen-containing ligands), which are combined with growth directing molecules of varied nature: amines, thiols, carboxylic acids, phosphines, and phosphine oxides. Given the soft and borderline Lewis acid nature of Cu^+^ and Cu^2+^, respectively (Fig. 6), proper coordination and hence control of the reactivity often requires the presence of thiols (*e.g.*, DDT) and/or phosphines (*e.g.*, TOP) – both of which have soft Lewis base behavior. Alkylamines (*e.g.*, OAm) are also often used, given their ability to form stable complexes with Cu^+^*via* L-type binding (*i.e.*, donation of an electron pair). Note that OAm can act simultaneously as reducing agent for copper, ligand, and solvent,^84^ not to mention its role in the release of reactive H_2_S when combined with elemental sulfur.^85^ DDT can also act as a mild reducing agent for copper.^86^ This aspect is of great relevance also in the synthesis of CuInS_2_ SNCs (see below).

In an early report on Cu_2_S SNCs, Wu *et al.* used a hot-injection approach whereby a solution of copper acetylacetonate and OA is injected into a hot solution of ammonium diethyldithiocarbamate in DDT and OA.^62^ The temperature of the reaction mixture was raised from 110 to 180 °C and the SNCs were obtained over the course of 20 min. As shown in a follow-up study, tuning the Cu : S feeding ratio allowed achieving control over the SNCs size from 2 to 6 nm in diameter. Xie *et al.* also proposed a hot-injection approach to obtain hexagonal CuS nanoplates by injecting an OAm-S solution into a mixture of CuCl, ODE, OA, and OAm at 180 °C.^57^ In their detailed study, the authors identified the crucial role played by the amine in coordinating Cu^+^ ions, proposing a Cu_*x*_(OAm)_*y*_(ODE)_*z*_Cl_*x*_ complex as intermediate for the nucleation and growth of the nanoplates, with oleyl ammonium oleate acting as a growth controlling species. Single-source precursors are also used in either heat-up or hot-injection thermal decomposition methods. These precursors include copper dithiocarbamates, xanthates, thiobenzoate, thiocyanate, and selenocyanate.^87,88^ Intriguingly, already in 2003, Larsen *et al.* reported on a solventless thermal decomposition of Cu-DDT to yield Cu_2_S nanorods and SNCs, even though no optical characterization was reported.^89^ Hsu *et al.* followed in these footsteps by reporting on nanodisks with varying Cu_2−*x*_S stoichiometry using both a solventless thermal decomposition approach and a heat-up, solvent-based technique, which allowed controlling the stoichiometry by tuning the Cu : S feeding ratio.^90^ Saldanha *et al.* also proposed a generalized heat-up strategy for the preparation of a library of Cu_2−*x*_S, Cu_2−*x*−*y*_S_*x*_Se_*y*_, and Cu_2−*x*−*y*_S_*x*_Te_*y*_ SNCs, where copper acetylacetonate and chalcogen powders (S, Se, Te) are dispersed in a DDT/OA mixture, followed by heating to 200–220 °C.^83^ A one-pot, heat-up approach was proposed by Xiao *et al.* too, who used different Se sources (selenurea, SeO, Se) to obtain Cu_2−*x*_Se nanomaterials with different morphology (spherical SNCs, nanorods, nanoplates).^91^ Similarly, Lord *et al.* obtained Cu_2−*x*_Se SNCs starting from copper acetylacetonate and diphenyl diselenide in OAm and ODE.^73^

The synthesis of pure Cu_2−*x*_Te SNCs is relatively less explored, and the quality of the SNCs in terms of size homogeneity is generally poorer. An approach that yields uniform cubic Cu_2−*x*_Te SNCs has been proposed by Li *et al.*, who used OAm, TOP, and TOPO as growth directing agents, while the Te precursor was prepared mixing TOP-Te and lithium bis(trimethylsilyl) amide in ODE.^92^ Nanorods and nanoplates were also obtained controlling the reaction parameters. Willhammar *et al.* later adopted this synthesis method and modified it into a one-pot heat-up approach, obtaining similar results in terms of SNCs quality.^79^ An intriguing alternative was proposed by Kriegel *et al.*, who prepared sacrificial CdTe SNCs to then convert them into Cu_2−*x*_Te SNCs.^80^ This approach leverages decades-worth of knowledge on the synthesis of quantum dots (QDs) with controlled size and morphology (*e.g.*, spheres, rods, tetrapods), bypassing the difficulties in adjusting these features directly during the synthesis of Cu_2−*x*_Te SNCs. While heat-up and hot-injection are arguably the most explored approaches for the preparation of Cu_2−*x*_E SNCs, sonochemical approaches^93^ and microwave-driven heating methods^94,95^ have also been proposed.

Given the widespread investigation on the use of Cu_2−*x*_E SNCs for biomedical applications (see below), both water-transfer methods and direct synthesis in aqueous media have been developed – in both cases, drawing inspiration from the protocols developed for QDs. For instance, van Oversteeg *et al.* proposed ligand exchange of DDT or TOPO molecules on the Cu_2−*x*_S SNCs surface for hydrophilic mercapto-acids (*i.e.*, 1-mercaptoundecanoic acid, 1-mercaptopropylic acid) or sulfide ions (S^2−^) from Na_2_S.^96^ Similarly to the approach followed by Saldanha *et al.*,^83^ exchange with thiolated polyethylene glycol (PEG) was also demonstrated by Marin *et al.* for CuS nanoplates.^87^ The same authors also demonstrated the straightforward synthesis of CuS SNCs in an aqueous solution of methoxy-terminated PEG molecules as growth controlling ligands.^97^ The so-obtained CuS SNCs display an easily modifiable surface chemistry, which makes them amenable to applications in different scenarios. As shown by Gan *et al.*, Cu_2−*x*_Se SNCs can also be prepared in aqueous environments using polyvinyl pyrrolidone (PVP) as growth directing agent at room temperature.^98^ Cu_2−*x*_S SNCs were also prepared directly in aqueous phase using citrate ions as growth controlling species and Na_2_S as the precipitating agent.^99^

#### 4.1.3. Applications

The use of Cu_2−*x*_E SNCs include energy conversion and storage, optoelectronics, catalysis, and photonics; yet, their use has been particularly investigated in the biomedical field.

##### Photovoltaics

The interest in Cu_2−*x*_S SNCs for photovoltaic applications^100^ is rooted in the discovery of the CdS/Cu_2_S heterojunction back in 1954.^101^ To that end, a mix of Cu_2_S SNCs and CdS nanorods were used to prepare a solar cell with 1.6% power conversion efficiency (PCE) (Fig. 5g).^62^ Cu_2−*x*_S SNCs featuring NIR LSPR too were used in photovoltaics, specifically to prepare conductive films later implemented in the architecture of a solar cell – again, together with CdS nanorods – to achieve PCE of 0.24%.^102^ CuS SNCs have also been used as hole-transporting-material in perovskite solar cells improving the cell's performance, but being limited in applicability by the large energy barrier between the VBs of perovskite and CuS.^103^ On the other hand, Cu_2_S SNCs have potential for the preparation of solution-processed electrodes in QD-sensitized solar cells (QDSSCs). The review by Burda and Zhao specifically tackles this application scenario of Cu_2−*x*_S SNCs, alongside their use as electrodes for Li ion batteries.^100^

##### Photocatalysis

The tunable optical properties of Cu_2−*x*_E SNCs make these nanomaterials suitable for photocatalysis too. Yet, their stability during operation is an issue for sustainable use of these materials in photocatalysis. Lie *et al.* showed that Cu_2−*x*_Se SNCs with controllable copper deficiency boost catalytic performance by >500× in luminol-H_2_O_2_ reactions, due to elevated hole density and ionic mobility. These features facilitate electron transfer processes that sustain H_2_O_2_ decomposition.^104^ Sun *et al.* showed that Au-Cu_2−*x*_S heterostructures act as effective photocatalyst through the study of rhodamine B as model pollutant,^105^ while Hoàng Ly *et al.* used CuS hollow nanospheres for photocatalytic reduction of CO_2_ into higher-value chemical species.^106^ A recent review by Li *et al.* thoroughly discusses the latest advancements and challenges in Cu_2−*x*_S photocatalysts specifically.^107^

##### Biomedicine

In biomedicine, Cu_2−*x*_E SNCs have been extensively studied as effective light-to-heat converters for photothermal applications. The possibility of obtaining LSPR in the NIR even at sizes well below 10 nm is an important differentiator compared to noble metal-based plasmonic nanomaterials – where NIR LSPR modes are obtained generally with bigger particles. The advantages of keeping the nanoparticle size small are two: (i) for some biomedical applications smaller particles are preferred due to their ability to elude certain biological barriers^108^ and (ii) smaller plasmonic nanoparticles feature dominant absorption *vs.* scattering, which is pivotal for many therapeutic and imaging applications. For instance, CuS SNCs were used for NIR-driven male mice sterilization, thanks to combined heating and reactive oxygen species (ROS) production (Fig. 5h).^63^ Similar materials and approach have been used for bacterial sterilization^109^ and to promote osteogenesis in periodontal therapy^110^−in the latter case, upon incorporation of the SNCs in a polymeric hydrogel. In the same vein, incorporation of CuS SNCs – alongside deferoxamine – in a biomimetic hydrogel yielded patches for photothermal therapy of infected diabetic wounds.^111^ Modification of CuS SNC surface with TAT and RGD peptides allowed Li *et al.* to achieve cell nucleus targeting at the tumor site, where NIR excitation resulted in localized photothermal therapy that prevented cancer recurrence.^112^ Neutrophil-erythrocyte membrane-coated hollow CuS SNCs were employed by Xue *et al.* to treat osteoarthritis *via* NIR-triggered local heating of joints.^113^ Copper selenide and telluride SNCs have been employed too in similar scenarios.^114–116^ Cu_2−*x*_Se SNCs and Au-Cu_2−*x*_Se heterostructures have also been used as contrast agents for *in vivo* photoacoustic imaging by Liu *et al.*^117,118^ The groups of Zhang reported on ultrathin CuS SNCs for photoacoustic imaging too,^119^ while Marin *et al.* showcased the first example of negative optical coherence tomography contrast agents in the form of CuS nanoplates.^87^

##### Others

A less common application scenario is the enhancement of upconversion emission *via* deposition of layers of luminescent NaYF_4_:Yb,Er nanoparticles and Cu_2−*x*_S SNCs separated by a buffer layer of MoO_3_ of controlled thickness (8 nm) (Fig. 5i). The >200-value reached by the enhancement factor was explained considering the increased scattering of the 980 nm excitation photons by the plasmonic film (at low power), as well as electron transfer from Cu_2−*x*_S SNCs to the upconverting nanoparticles upon two-photon excitation of the sulfide species (at high power).^64^ Worth highlighting is also the work by Zhang *et al.*, who demonstrated the potential of these materials for sensing, showing that aggregates of Cu_2−*x*_S SNC can be effectively used in surface-enhanced Raman scattering (SERS).^120^

### 4.2. Ternary compounds and beyond

#### 4.2.1. General considerations, structure, and optical properties

Ternary copper chalcogenide SNCs have general formula Cu_*x*_M_*y*_E_*z*_, where M = In, Ga, Al, Fe, Bi, Sb, Sn, Ge, Cr, Zn. These materials can usually retain their crystal structure even when strong deviations from the theoretical stoichiometry occur. They can be prepared in alloyed forms, such as CuIn_1−*x*_Ga_*x*_S_2_ or Cu_3_(Sb_1−*x*_As_*x*_)S_4_ or they can be alloyed with other binary semiconductors, such as CuInS_2_–ZnS (generally indicated as Cu–In–Zn–S). These compositions are often indicated as quaternary – or even higher-order – compounds, and they often have intermediate properties compared to the individual components. Indeed, this type of alloying is usually a strategy employed to fine tune the absorption and emission of photoluminescent SNCs between the two “extreme” properties featured by the two components.

Below, we provide a discussion on structure–property relationships in multinary Cu-based SNCs broken down by compound families: Cu-III-E, Cu-IV-E, and Cu-V-E (where III, IV, and V indicate the groups the metals belong to), Cu–TM–E (TM = transition metal), and quaternary materials.

##### Cu-III-E compounds

*CuInE*_*2*_ *and CuGaE*_*2*_. CuInE_2_ and CuGaE_2_ are families of p-type semiconductors. Together with CuGaE_2_, AgInE_2_, and AgGaE_2_ are materials that have been investigated and/or are currently successfully employed for the preparation of photovoltaic devices including thin-film solar cells and QDSSCs (see below). Their properties are dominated by a marked tolerance against deviations from both cation and anion stoichiometries.

**Fig. 7:**
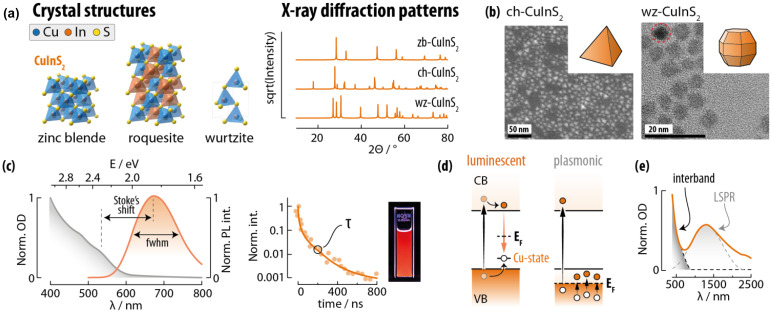
Copper indium sulphide SNCs as representatives of the Cu-III-E family. (a) Unit cell and simulated XRPD patterns of the three CuInS_2_ polymorphs. (b) TEM images and corresponding typical morphology of CuInS_2_ in the chalcopyrite (ch-CuInS_2_) and wurtzite (wz-CuInS_2_) crystal structure. TEM images reprinted with permission from ref. 121 (Copyright 2010, American Chemical Society) and ref. 122 (Copyright 2017, American Chemical Society). (c) Typical extinction and emission spectrum (left), photoluminescence decay curve (centre), and photo under UV illumination (right) of a CuInS_2_ SNCs dispersion. Adapted with permission from ref. 123 Copyright 2019, American Chemical Society. (d) Energy level schemes and corresponding transitions that underpin the optical properties of luminescent and plasmonic CuInS_2_ SNCs. (e) Extinction spectrum of plasmonic CuInS_2_ SNCs. Adapted with permission from ref. 124, Copyright 2012, American Chemical Society.

CuInS_2_ is arguably the most studied ternary copper chalcogenide for the preparation of SNCs. This material crystallizes in three polymorphs: roquesite (*I*4̄2*d*; the thermodynamically stable phase akin chalcopyrite), wurtzite (*P*6_3_*mc*), and zinc blende (*F*4̄3*m*) (Fig. 7a).^15^ Chalcopyrite and wurtzite are the polymorphs usually stabilized at the nanoscale (ch-CIS and wz-CIS). Most publications on CuInS_2_ SNCs deal with chalcopyrite phase, although chalcopyrite can hardly be discerned from zinc blende simply *via* X-ray powder diffraction (XRD) – the prime technique employed for phase attribution for these materials. In this phase, Cu^+^ and In^3+^ ions occupy the crystal sites in an ordered fashion, while in the other two polymorphs the cations are randomly distributed over the cation sublattice. Another difference between phases lies in the morphology of the corresponding SNCs: ch-CIS takes up a tetrahedral habitus, while wz-CIS is generally assumed quasi-spherical (although actually having more complex morphologies such as truncated octahedron; Fig. 7b). Note that the chalcopyrite crystal phase is recurring in the chemistry of multinary Cu-chalcogenides, and it can be seen as a derivation of the zinc blende crystal structure (see below).

CuInS_2_ has a direct bandgap of approximately 1.5 eV, absorption coefficients in the 10^5^ cm^−1^ range, and a Bohr exciton radius of 4.1 nm. The interest around CuInS_2_ SNCs stems from the visible and NIR photoluminescence that these nanomaterials display, with PLQY values at times higher than 90%.^125^ Moreover, CuInS_2_ SNCs feature an absorption cross section of 1.8 × 10^−16^*a*^3^ cm^2^ (where *a* is the SNC radius): On par with typical values of for CdSe QDs.^126^ As such, CuInS_2_ SNCs have brightness values comparable to those of CdSe QDs, while lacking more toxic Cd^2+^ in their composition. However, the optical properties of CuInS_2_ SNCs are drastically different from the ones of binary QDs: (i) the excitonic absorption peak is poorly defined in the absorption spectrum, (ii) the emission peak is broad (fwhm > 300 meV) and (iii) characterized by a large Stokes shift (300–500 meV), (iv) the characteristic photoluminescence decay time is long (100–500 ns) (Fig. 7c). To explain these characteristics, several models have been proposed, which invoke electronic transitions of different types: donor–acceptor-pair (DAP), band-to-band, free-to-bound, and self-trapped excitons. In a recent review by the Wang group, the debate over the origin of the luminescence in these nanomaterials is described in detail.^15^ Albeit no consensus has been reached yet, the community lean towards the free-to-bound model, where the CuInS_2_ emission stems from the recombination between a free/unbound electron in the CB and a hole localized at a copper-related trap state just above the VB (Fig. 7d). This attribution is also supported by the striking similarities observed with the emission of Cu^+^-doped ZnS and CdS QDs.^127^ Intriguingly, the emission of CuInS_2_ SNCs can be tuned by adjusting the Cu : In ratio.^128^ Specifically, within a certain range of off-stoichiometry, Cu-deficient SNCs display blue-shifted emission and an increased PLQY. The origin of the effect has not been clearly identified, with some authors claiming the involvement of copper vacancy-related states (V_Cu_) in the radiative recombination process.^129^ However, ensuring consistent particle size when varying the Cu:In ratio is difficult, thus complicating the study of off-stoichiometry effects. To that end, other approaches for tuning and improving the optical properties of CuInS_2_ SNCs entail:

(i) Control of the SNC size to harness the quantum confinement effects.

(ii) The growth of core/shell structures with type-I band alignment using ZnS or CdS as shell material. In the case of ZnS, the reactivity of the ZnS precursors^125^ and the surface chemistry of the core SNCs can be tuned to induce partial Cu^+^–Zn^2+^ cation exchange (hence forming a gradient shell) or exclusively ZnS overgrowth.^130^

(iii) Alloying with other elements such as Ag^+^ to substitute Cu^+^ and Ga^3+^ or Al^3+^ (albeit with more difficulty due to the ionic radius mismatch) for In^3+^.^131–135^ This strategy enables spanning a broad spectral range, since the obtained SNCs can show emission spanning from the blue all the way to the NIR (approx. 450–1000 nm). Alloying with Zn^2+^ is also an explored avenue.^136,137^ This strategy exploits the miscibility over the whole composition range of CuInS_2_ and ZnS, due to the structural similarities between the two materials.

Besides, photoluminescence, CuInS_2_ SNCs can also sustain LSPR modes (Fig. 7e). The Rosenthal group was the first to report on plasmonic chalcopyrite CuInS_2_ SNCs.^124^ They were synthesized with highly reactive bis(trimethylsilyl) sulfide, which induced the incorporation of metal vacancies – due to the rapid SNC formation – that support LSPR. An *N*_H_ = 2.6 × 10^20^ cm^−2^ was found analyzing the extinction spectrum according to the Drude formalism. Subsequently, the groups of Swihart^138^ and Ghosh^139^ showed that plasmonic CuInS_2_ SCNs are obtained in the presence of excess Cu^+^ in the structure. This photoluminescence-plasmonic duality is an intriguing feature of CuInS_2_ SNCs, which highlights the versatility of this type of nanomaterial.

CuInSe_2_ is similar in structure and properties to CuInS_2_.^140,141^ CuInTe_2_ is instead mainly found in the chalcopyrite polymorph. In bulk, CuInSe_2_ and CuInTe_2_ have bandgaps of 1.04^140,142,143^ and 1.06 eV,^144^ with absorption coefficients in the 10^5^ cm^−1^ range. As their bandgap, the emission of these materials in shifted more towards the NIR range than CuInS_2_. The means of controlling the optical properties of these SNCs are also the same as the ones described for CuInS_2_ SNCs.^142,145–147^ The emission of CuInSe_2_ SNCs, for instance, can be effectively tuned from approx. 800 to 1400 nm by means of alloying with Ag^+^ and Zn^2+^, followed by overgrowth of a ZnS shell for maximized PLQY.^148^ Like in the case of binary Cu_2−*x*_E SNCs, the telluride of the CuInE_2_ family are underexplored compared to sulfides and selenides, and are often reported in alloys with lighter chalcogenides.^146^

Similar considerations can be made for the CuGaE_2_ SNC family. CuGaS_2_, CuGaSe_2_, and CuGaTe_2_ all crystallize in the chalcopyrite crystal phase and have direct bulk bandgaps of 2.43, 1.68, and 1.2–1.9 eV, respectively. The optical properties of SNCs made of these materials (as well as the strategies proposed to tune them) resemble those reported for CuInE_2_ SNCs, yet they are more shifted at higher energy (*i.e.*, shorter wavelengths).^149–152^

*CuAlE*_*2*_. Closely related to two families of semiconductors mentioned above are CuAlS_2_ and CuAlSe_2_. Both these semiconductors crystallize in the chalcopyrite phase and have wide bandgaps of approximately 3.5 and 2.7 eV, respectively.^153–156^ Although core-only CuInS_2_ SNCs do not appear to feature appreciable photoluminescence, the Pandey group showed that preparation of type-II core/shell SNCs using CdS as shelling material results in tunable emission in the visible-NIR range.^157^ The observed emission was explained in light of delocalization of the exciton in the shell, also given the dependence of its energy on the shell thickness. The same group also prepared CuAlS_2_-ZnS heterostructures, observing a similar charge delocalization, thus achieving bandgap values in the 1.5–2.0 eV range despite the fact that both core and shell materials have bandgaps in the UV-blue region.^158,159^ Cu–Al–S/ZnS SNCs were later prepared by Hansen *et al.*, obtaining blue-emitting species with PLQY as high as 18%. The study highlighted the presence of a complex, defect-rich electronic structure of the SNCs, which was induced also by deliberate synthesis of highly Cu-defective nanomaterials. Combination of theoretical calculations and experimental observations suggested that the SNC cores were actually made of an ordered defect compound with composition CuAl_5_S_8_. Intriguingly, the optical properties of CuAlS_2_ SNCs were also optimized *via* a combination of design-of-experiments and density functional theory (DFT) calculations by Baum *et al.*^160^ In that study, also NIR absorption features were observed in some samples, suggesting the existence of resonances in CuAlS_2_−which are currently underexplored.

##### Cu-IV-E compounds

**Fig. 8:**
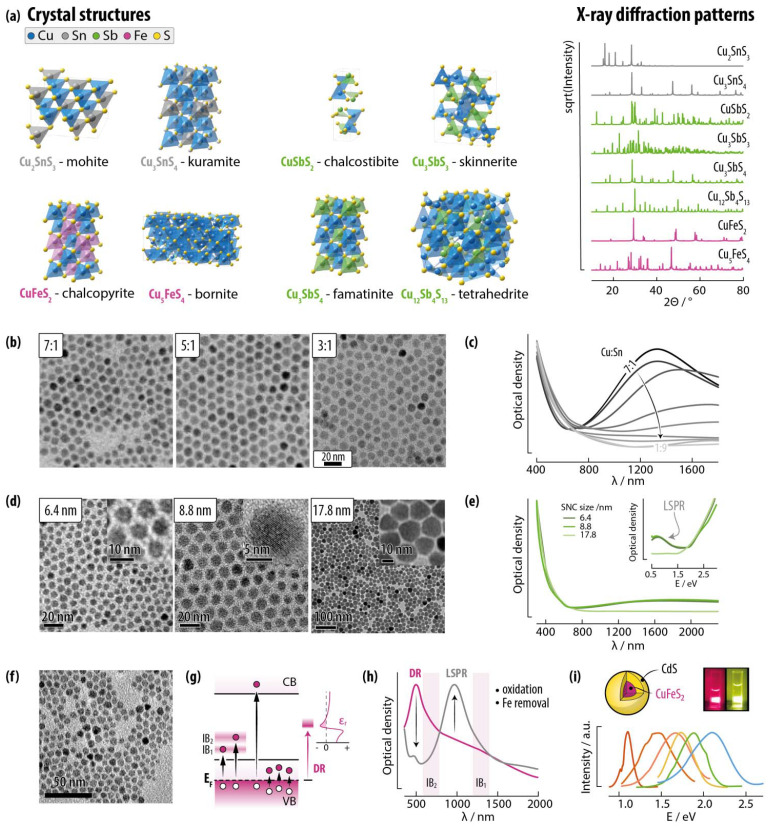
Copper tin sulphide, copper antimony sulphide and copper iron sulphide SNCs as representatives of other ternary copper chalcogenides. (a) Unit cell and simulated XRPD patterns of the most common compositions reported for Cu–Sn–S, Cu–Sb–S, and Cu–Fe–S SNCs. (b) Representative TEM images and (c) extinction spectra of Cu_2_SnS_3_ SNCs prepared with different Cu:Sn ratios. Reproduced with permission from ref. 162, Copyright 2015, American Chemical Society. (d) Representative TEM images and (e) extinction spectra of Cu_12_Sb_4_S_13_ SNCs of different sizes. Adapted with permission from ref. 167, Copyright 2013, American Chemical Society. (f) TEM image of CuFeS_2_ SNCs. Reprinted with permission from ref. 168, Copyright 2021, American Chemical Society. (g) Electronic structure typical of Cu–Fe–E SNCs including intrabandgap states conducive of dielectric resonances. (h) Effect of oxidation and Fe removal on the optical properties of CuFeS_2_ SNCs. Adapted with permission from ref. 169, Copyright 2021, American Chemical Society. (i) Photoluminescence of CuFeS_2_/CdS core /shell SNCs of different sizes. Adapted with permission from ref. 170 Copyright 2016, American Chemical Society.

*Cu*–*Sn*–*E*. In this family of semiconductors, the most common compounds reported in the form of SNCs are Cu_2_SnS_3_ (mohite, *Cc* – although other polymorphs are also known)^161^ and Cu_3_SnS_4_ (kuramite, *I*4̄2*m*) (Fig. 8a). A metastable wurtzite structure is also reported in the literature only for SNCs, and Cu–Sn–S SNCs in the kesterite phase were obtained too.^162,163^ Cu_2_SnSe_3_ crystallizes in the cubic *F*4̄3*m* (zinc blende)^164^ or monoclinic *Cc* (okruginite)^165^ structures. For an overview on Cu–Sn–Se SNCs, the reader is directed to ref. 164 and 166. Such richness of phases obtainable at the nanoscale is testament of the versatility of nanomaterials, where ligand and precursor nature and concentration act synergistically to determine the properties of the SNCs. SNCs of Cu–Sn–E display strong light absorption capabilities as well as LSPR modes supported by metal vacancies in the structure. This observation was corroborated by a study by the Swihart group, who showed that the strength of the resonance decreased with increasing Sn content in the SNCs (Fig. 8b and c).^162^

*Cu*–*Ge*–*E*. Among this family of materials, Cu_2_GeS_3_ and Cu_2_GeSe_3_ are the most studied as far as SNC production is concerned. Cu_2_GeS_3_ (bulk direct bandgap of 1.5–1.6 eV) is reported to crystallize in bulk and in SNC form in the *Cc* space group.^165^ Yet, in SNCs it crystallizes also in cubic zinc blende-like (*F*4̄3*m*) or metastable hexagonal wurtzite-like (*P*6_3_/*mc*) phases.^171,172^ An orthorhombic (*Pmn*2_1_) phase has been observed for Cu_8_GeS_6_ SNCs.^173,174^ Also for these nanomaterials, LSPR features have been observed, besides interband electronic transitions.^171^

##### Cu-V-E compounds

*Cu*_*3*_ *BiS*_*3*_. Although some alternative compositions of Cu–Bi-based chalcogenide nanomaterials are reported,^175^ Cu_3_BiS_3_ is by far the most prevalent.^176^ Cu_3_BiS_3_ is a p-type semiconductor with bulk bandgap of 1.5 eV that crystallizes in the wittichenite phase (orthorhombic, *P*2_1_2_1_2_1_). SNCs of this material are generally of interest due to their strong light absorption capabilities in the visible. Yan *et al.* also observed the presence of a NIR-centered absorption feature, which was ascribed to an LSPR mode.^177^ Similar features in the NIR are observed in the study by Chakraborty *et al.* and Ye at al.,^178^ while Paul *et al.* even recorded both transversal and longitudinal LSPR modes in alloyed Cu_3_BiS_3−*x*_Se_*x*_ nanorods obtained through cation exchange from Bi_2_S_3−*x*_Se_*x*_.^179^ Note that Cu_3_BiSe_3_ can also be directly obtained, liked showed by Du *et al.*, who synthesized hydrophilic SNCs of this material in ethylene glycol and observed LSPR modes centered above 1200 nm.^180^

*Cu*–*As*–*S and Cu*–*Sb*–*S*. These families of materials include CuSbS_2_ (chalcostibite, *Pnma*), Cu_3_AsS_4_ and Cu_3_SbS_4_ (luzonite and famatinite, *I*4̄2*m*), Cu_12_As_4_S_13_ and Cu_12_Sb_4_S_13_ (tennantite and tetrahedrite, *I*4̄3*m*).^181–185^ Also Cu_3_SbS_3_ (skinnerite, *P*2_1_/*c*) has been reported (Fig. 8a).^186^ These materials have various bandgaps from 1.0 to 2.0 eV and absorption coefficients in the 10^5^ cm^−1^ in the visible range.^185,187,188^ Ternary chalcogenides of As and Sb are sometimes prepared in alloyed forms, given their good miscibility.^181^ Note that also Cu–As–Se and Cu–Sb–Se are reported, but we decided to omit them from this review given the limited number of reports on SNCs made of these materials.^189,190^ Aside from typical photo-induced interband electron transitions, it is worth mentioning the observation in Cu–Sb–S SNCs of a possible NIR LSPR by van Embden *et al.* (Fig. 8d and e)^167^ and nonlinear optical absorption, accompanied by ultrafast carrier dynamics, by Zhang *et al.*^188^

##### Cu–TM–E

*Cu*–*Fe*–*E*. In these family of semiconductors,^191^ CuFeS_2_ (Fig. 8f) and CuFeSe_2_ are among the most studied. CuFeS_2_ is often reported as an n-type semiconductor that crystallizes in the chalcopyrite phase with a bulk bandgap of 0.6 eV. The Cu–Fe–S system (often indicated as Cu_*x*_Fe_*y*_S_*z*_) is very rich in composition, and includes the orthorhombic Cu_5_FeS_4_ structure (bornite, *Pbca*) (Fig. 8a). CuFeSe_2_ has a different, yet still tetragonal, crystal structure (eskebornite; *P*4̄2*c*) with a bulk bandgap varying considerably from one report to another in the 0.16–0.6 eV range.^192–194^

The presence of iron in the composition of these materials results in a unique electronic structure, since the empty 3d orbitals of this metal introduce intermediate band (IB) states, which partake in electronic transitions (Fig. 8g). Moreover, this electronic structure leads to negative permittivity values within specific wavelength ranges that support so-called quasi-static dielectric resonances (DRs).^195^ In fact, various recent studies observed both DR and more common LSPR contributions in the extinction spectra of both chalcopyrite and bornite SNCs.^168,196–198^ These two resonant contributions are fundamentally different in nature (Fig. 8h) and their relative contributions to overall extinction can be modulated *via* controlled oxidation. In a follow-up study employing transient absorption spectroscopy, Kuszynski *et al.* found that the amount of Fe in the bornite SNCs correlates with the position of the IB, also identifying its central role in the relaxation dynamics of both hot holes and exciton carriers.^199^

Remarkably, CuFeS_2_ SNCs have been used also as cores for photoluminescent core/shell nanomaterials (Fig. 8i). Bhattacharyya and Pandey showed that, similarly to CuAlS_2_, core-only CuFeS_2_ SNCs are not luminescent, but the growth of a gradient CdS shell activates their luminescence.^170^ The emission could be tuned in the visible range controlling the size of the core in the core/shell structure; however, the actual emission mechanism was not definitively interpreted. Vlasovets *et al.* also showed photoluminescence in Cu–Fe–S SNCs incorporated into a polymeric film,^200^ while CuAl_*x*_Fe_1−*x*_S_2_/ZnS were observed to have tunable emission in the visible-NIR range with large Stokes shifts.^201^

*Others*. Other less-reported SNCs are made of materials including Cu_2_MoS_4_ (*P*4̄2*m*),^202^ CuCo_2_S_4_ (*Fd*3̄*m*),^203^ Cu_3_VS_4_ (*P*4̄3*m*),^204^ CuCrS_2_ (*R*3*m*), CuCrSe_2_ (*R*3*m*),^205^ CuCr_2_Se_4_ (*Fd*3̄*m*),^206^ Cu_2_MnS_2_ (*Fd*3̄*m*). The optical properties of these SNCs are dominated by interband photon absorption.

##### Quaternary compounds

The definition of quaternary Cu-chalcogenide compounds can be blurry. In fact, many claimed quaternary materials can be considered alloys of other (ternary and/or binary compounds), with their electronic and optical properties falling in between the ones of the constituent materials. Moreover, the higher the number of the combined elements, the higher the chance of introducing disorder in the structure. Here, we consider as quaternary compounds those with well-defined stoichiometries. There are many possible such materials, and here we select a few that are well studied. Among those, quaternary semiconductors that include Zn^2+^ and/or Sn^4+^ are the most prevalent.

**Fig. 9:**
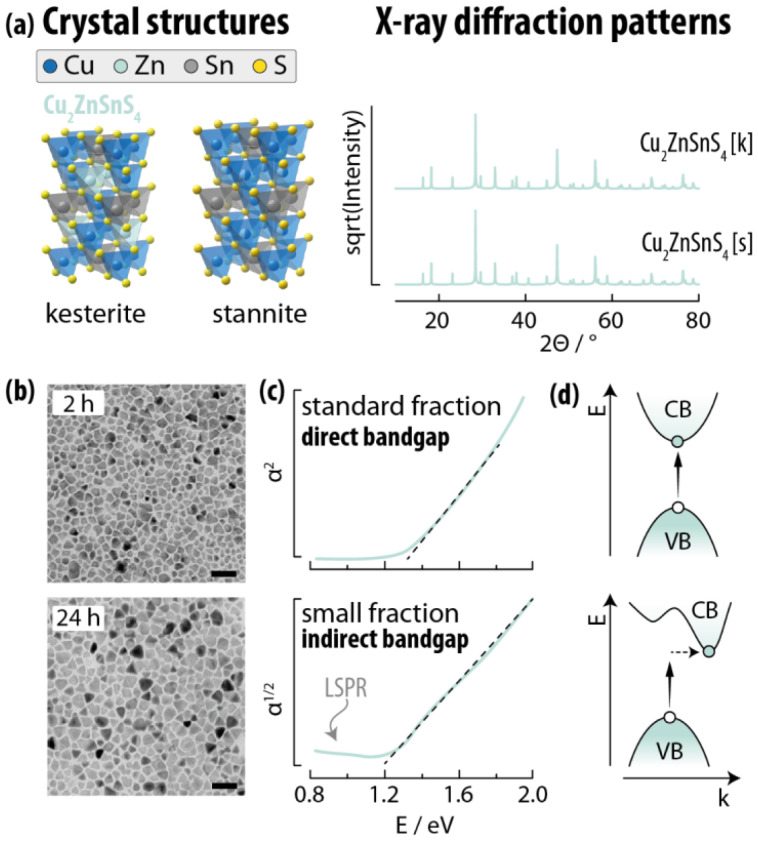
Copper zinc tin sulphide SNCs as representatives of quaternary copper chalcogenides. (a) Unit cell and simulated XRPD patterns of the two most common compositions reported for Cu_2_ZnSnS_4_ SNCs. (b) TEM images of kesterite SNCs at different reaction times, alongside (c) the extinction spectra of SNCs obtained by precipitating smaller and larger SNC populations. Scale bars are 50 nm. Adapted with permission from ref. 209, Copyright 2015, American Chemical Society. (d) Band alignment of a direct (top) and indirect (bottom) bandgap semiconductor.

With its 1.0–1.5 eV direct bandgap,^207^ good stability, and high absorption coefficient,^208^ Cu_2_ZnSnS_4_ is used for the preparation of solar cells given the fact that it is solely made of Earth-abundant elements. In its bulk form, Cu_2_ZnSnS_4_ crystallizes in the kesterite (*I*4̄) and stannite (*I*4̄2*m*) crystal phases (Fig. 9a), both closely related to chalcopyrite. Indeed, they are virtually distinguishable *via* XRD characterization alone, especially at the nanoscale. Kesterite is the most common Cu_2_ZnSnS_4_ crystal structure in SNCs. To that end, Collord and Hillhouse reported on kesterite SNCs with different compositions and sizes (Fig. 9b–e).^209^ Intriguingly, the authors found that the optical properties of larger SNCs are better described by a direct electronic transition, while small SNCs show an indirect bandgap with and sustain LSPR modes (Fig. 9c). A wurtzite phase has been observed too in SNCs. Initially reported by Mainz *et al.* as a transitional phase towards the formation of stable kesterite SNCs,^210^ some authors later reported on stable wurtzite SNCs likely obtained through the initial formation of Cu_2−*x*_S and/or Cu_2_SnS_3_ seeds.^211–213^ The interest around wurtzite phase Cu_2_ZnSnS_4_ stems from the stoichiometric flexibility of this material – and hence high tunability of its optical properties – given the random distribution of the metals over the cation sublattice.

The sustainability of Cu_2_ZnSnS_4_ was further boosted in a recent study by Jones *et al.*, where the authors demonstrated scalability of the optimized synthesis of these SNCs, and gauged a >30% environmental impact reduction through life-cycle assessments of the materials.^214^

Other quaternary SNCs include, among others, those made of Cu_10_Co_2_Sb_4_S_13_, Cu_10_Zn_2_Sb_4_S_13_, Cu_10_Ni_1.5_Sb_4_S_13_, Cu_2_ZnGeS_4_, Cu_2_CdGeS_4_, Cu_2_CoGeS_4_, and Cu_2_ZnGeSe_4_.^215–220^

#### 4.2.2. Synthesis

**Fig. 10:**
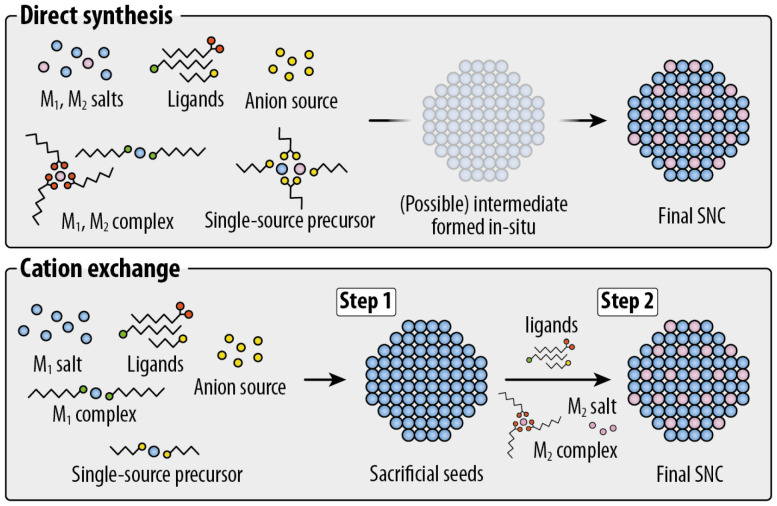
Scheme of direct synthesis and cation exchange approaches.

The preparation of multinary Cu-based chalcogenide SNCs has been explored through a multitude of approaches. Like in the case of binary Cu_2−*x*_E SNCs, hot-injection and heat-up approaches are the most commonly employed ones, alongside solvo- and hydrothermal, sonochemical, microwave-driven, as well as microfluidic-based methods. In addition, to these “direct” methods, multi-step protocols have been developed, which are based on cation exchange processes. Notably, also most direct methods occur through the formation of SNCs of a specific composition followed by slower cation exchange or cation incorporation processes to achieve the final material. However, for the sake of the discussion, these two approaches are presented independently (Fig. 10), depending on whether the cation exchange is kept by design clearly separated in the synthesis.

##### Direct approaches

The fundamental challenge in the synthesis of multinary compounds in direct approaches lies in the different nature of the elements that compose the material. In that regard, let us consider the case of CuInS_2_. The metal ions in this material are Cu^+^ and In^3+^, which display soft and hard Lewis acid behavior, respectively (Fig. 6). As such, they interact differently with different molecules, them being amines, carboxylate, thiolate, phosphines, or phosphine oxides. Hence, different ligands – at carefully controlled concentrations – should be introduced in the reaction environment to form *in situ* metal precursors with balanced reactivity. This is necessary to achieve the targeted multinary SNC rather than two populations of, in this case, Cu_2−*x*_S and In_2_S_3_ SNCs.

An alternative is the use of single-source precursors that contain both metals in the right stoichiometric proportion (here, equimolar Cu and In amounts) alongside the chalcogen. The thermal decomposition of the precursor results in the simultaneous release of the elements in the right proportion, thus favoring the formation of the multinary compound. This approach seems promising on paper and thus attracted most of the attention in the early days of research on these SNCs. However, the time-consuming preparation of the precursors, their often air-sensitive nature, and our advanced understanding of wet chemistry approaches for nanoparticle synthesis have favored other, more user-friendly methods.

In hot-injection methods, metal precursors (salts and/or complexes) are mixed with a solution containing one or more species of ligands, the slurry is then heated to a target temperature generally >100 °C under vacuum to promote the precursor dissolution and *in situ* formation of metal complexes (*e.g.*, metal carboxylates, metal thiolates). The temperature is then increased to a target value and a solution containing the chalcogen (in the form of elemental S, Se, or Te or a thermally labile chalcogen-containing molecule) is rapidly injected into the metal solution. CuFeSe_2_ nanocubes have been prepared by Wang *et al.* following this type of approach and using diphenyl selenide as chalcogen precursor.^221^ The Dennis group reported the synthesis of bornite SNCs through a similar approach with OAm-S as the chalcogen solution, which was gradually injected over the course of 5 min at 180 °C.^169^ The authors also observed that fast injection of the chalcogen solution in a glovebox led to a better controlled SNC size. Even quaternary SNCs can be prepared with this approach, as shown by Zhu *et al.*, who synthesized Cu_2_CoGeS_4_ SNCs, also demonstrating the possibility of replacing effortlessly Co for other TM such as Fe, Ni, Cd, and Mn.^219^ Similar approaches are reported for most of the SNC families described above. Very recently, in an elegant study Chen *et al.* reported on a generalized strategy for the preparation of highly monodispersed quaternary wurtzite Cu-based selenide SNCs *via* injection of a diphenyl diselenide solution in a mixture of OAm, DDT, and the metal precursors.^222^

In a heat up approach, all the species are mixed together and the mixture is gradually heated to a target temperature. Lower-reactivity chalcogen species are generally used in this approach, to avoid uncontrolled SNC growth already at low temperature. In the case of CuInS_2_ SNCs, the heat up approach has been perfected over the years: The preferred method entails the use of DDT in large excess – sometimes together with some OA and diluted with ODE – in the presence of CuI and In-acetate. Upon increasing the temperature, yellow metal-thiolates are formed above 100 °C, which start decomposing around 180 °C to give ch-CIS QDs. Note that various Cu^+^ precursors can be used, including Cu^2+^ salts. Use of divalent copper is also possible, despite the +1 oxidation state of copper in practically all multinary Cu-based semiconductors, thanks to the reducing capabilities of some molecules such as DDT or amines (*e.g.*, OAm).^215^ Moreover, the copper counterion can partake in the SNC growth, remaining incorporated in the crystal structure too.^223^ Quaternary, Cu_2_ZnGeS_4_ SNCs were prepared *via* a heat up approach by Fan *et al.*^217^ Yet, as mentioned previously, the growth mechanism was shown to be step-wise, with an initial formation of Cu_1.75_S SNCs, followed by rapid incorporation of Zn^2+^ to yield a Cu_*x*_Zn_*y*_S wurtzite phase, followed by gradual Ge^4+^ incorporation to give Cu_2_ZnGeS_4_ SNCs in an orthorhombic crystal phase.

##### Cation exchange approaches

The advantages of using cation exchange approaches are several: (i) a single batch of sacrificial (often Cu_2−*x*_E) SNCs can be used to obtain wildly different compositions, (ii) crystal structures otherwise unachievable *via* direct methods can be stabilized for a given composition depending on the structure of parent SNCs, and (iii) upon controlling the reactivity of the exchanging ion solution, unique heterostructures can be unlocked.^224,225^ The clear drawback of this approach is its time-consuming and less-straightforward nature compared to, *e.g.*, heat-up approaches. However, in the case of Cu-chalcogenide SNCs, cation exchange methods are possibly unrivalled in terms of flexibility and degree of control on the optical properties of the produced SNCs.^226^

The kinetics of cation exchange processes are tuned by adjusting the composition of the cation exchange solution. Specifically, the ligand should promote extraction of the ion in the sacrificial SNC rather than too markedly stabilizing in solution the ion to be pushed in the structure. Because Cu_2−*x*_E SNCs are most often the sacrificial species, thiols and phosphines (soft Lewis bases) are used to promote Cu^+^ extraction. For example, Akkerman *et al.* showed that low chalcocite Cu_2−*x*_S SNCs prepared *via* a thiourea-mediated heat up approach can be used as sacrificial species for preparing CuInS_2_ SNCs.^227^ Incorporation of In^3+^ was achieved in the presence of TOP at 120 °C, achieving wurtzite CuInS_2_ SNCs. An additional cation exchange process with Zn^2+^, OAm, and octylamine (OcAm) led to incorporation of this divalent cation and a consequent enhancement of the photoluminescence intensity of CuInS_2_/ZnS with a gradient ZnS shell.^227^ Shamraienko *et al.* treated instead CuSe nanoplates with a Zn^2+^ and Sn^2+^ solution in OAm/OcAm in the presence of excess TOP, to obtain Cu_2_ZnSnSe_4_ nanoplates.^78^ One should also consider the oxidation state of the cation to be exchanged, thus promoting ligand-free incorporation. To that end, Liu *et al.* showed that Sn^2+^ can be effectively incorporated in CuS SNCs to prepare Cu_3_SnS_4_ SNCs, while Sn^4+^ could only be incorporated to yield Cu_2_SnS_3_ SNCs if DDT was also added in the dispersion.^228^ The authors explained this effect considering the S–S reducing capabilities of Sn^2+^, which promoted conversion of the crystal structure from covellite to kuramite.

#### 4.2.3. Applications

Similarly to Cu_2−*x*_E SNCs, the main application fields for multinary Cu-based SNCs are photovoltaics and optoelectronics, photocatalysis, and biomedicine.

##### Photovoltaics & optoelectronics

**Fig. 11:**
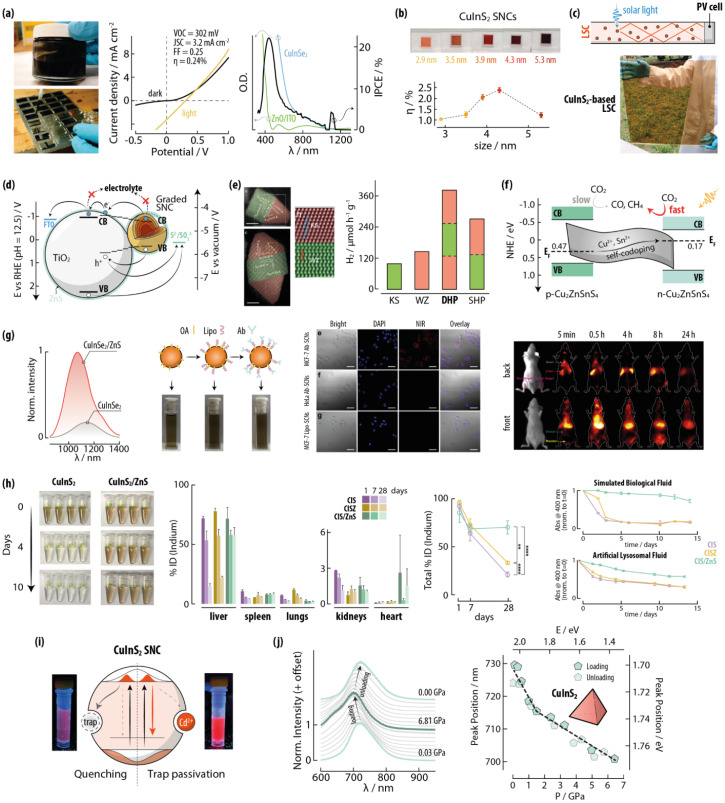
Applications and case studies for multinary Cu-based SNCs. (a) Photo of a CuInSe_2_ SNC ink and its deposition onto glass substrates (left), alongside a representative current-vs-potential curve under dark conditions (black) or light exposure (yellow) of a corresponding photovoltaic device (centre) and its ICPE spectrum (right). Adapted with permission from ref. 229, Copyright 2008, American Chemical Society. (b) Photo of ITO/CuInS_2_ SNCs photoanodes (top) and power conversion efficiency of the corresponding solar cells (bottom) for different SNC sizes. Adapted with permission from ref. 244, Copyright 2014, American Chemical Society. (c) Scheme showing the working principle of a LSC (top) alongside a photograph of a 30 × 30 × 0.7 cm LSC waveguide of CuInS_2_ SNC in PMMA. Reprinted with permission from ref. 245, Copyright 2019, John Wiley and Sons. (d) Scheme showing the approximate band alignment and electron transfer processes (black lines) in the graded CuInSe_2_/(CuInSe_*x*_S_1−*x*_)_5_/CuInS_2_ SNCs and TiO_2_ photoanode. Adapted with permission from ref. 249, Copyright 2021, Elsevier. (e) TEM images of kesterite–wurtzite (red-green) Cu_2_ZnSnS_4_ single (top) and double (bottom) homojunctions, alongside a high-resolution image showing the atomic arrangement in the two polymorphs. Scale bars are 5 nm. Adapted with permission form ref. 250, Copyright 2022, Springer. (f) Scheme showing the energy levels of p- and n-type Cu_2_ZnSnS_4_ SNCs and their use in CO_2_ photoreduction. Adapted with permission from ref. 251, Copyright 2022, American Chemical Society. (g) Use of CuInSe_2_/ZnS SNCs coated with phospholipids and antibodies for *in vivo* tumour imaging. From left to right: The emission spectra, functionalization process, study of cell interaction, and NIR images in nude mice are reported. Adapted with permission from ref. 252, Copyright 2020, Elsevier. (h) Study of the cytotoxicity profile of CuInS_2_-based SNCs. From left to right: photos of CuInS_2_ and CuInS_2_/ZnS SNCs in simulated biological fluid at different days, degradation of CuInS_2_ (CIS), Cu–In–Zn–S (CISZ), and CuInS_2_/ZnS (CIS/ZnS) SNCs monitored *via* optical absorption spectroscopy, organ-specific distribution (as % of In^3+^ initial dose) and summed In^3+^ content found in the body for CIS, CISZ, and CIS/ZnS. Adapted with permission from ref. 253, Copyright 2020, American Chemical Society. (i) Scheme representing the effect of Cd^2+^ occupying surface traps in CuInS_2_ SNCs responsible for photoluminescence quenching. Adapted with permission from ref. 254, Copyright 2025, American Chemical Society. (j) Emission spectrum of CuInS_2_ SNCs during a loading/unloading cycle in a diamond anvil cell, with the corresponding calibration dataset using peak maximum position as the manometric readout feature. Adapted with permission from ref. 255, Copyright 2023, John Wiley and Sons.

Photovoltaic cells with Cu(In,Ga)(S,Se)_2_ absorbing layer are commercially available. These are known as CIGS solar cells and display some of the highest absorption coefficients among photovoltaic devices. Therefore, SNCs with such compositions are used to prepare inks for the fabrication of printable photovoltaic devices (Fig. 11a).^229–231^ Intriguingly, plasmonic CuInS_2_ SNCs were shown to be more effective than their non-plasmonic counterpart for this application. This is because of the absorption enhancement ensured by the partial overlap between the interband absorption and LSPR bands (Fig. 7e).^124^ Along the same lines, inks of Cu_2_ZnSnS_4_ (CZTS) and Cu_2_ZnSnSe_4_ (CZTSe) SNCs have been proposed to prepare printable devices,^232–235^ and other compositions have been explored too. As a matter of fact, the first reported synthesis of CZTS and CZTSe SNCs was reported by Guo *et al.*, who demonstrated the suitability of those SNCs dispersion for preparing solar cells.^232^ Indeed, the interest around these materials stems from a theoretical 32% PCE value of thin-film solar cells based on kesterite.^236^ The film preparation techniques that guarantee better film homogeneity and thickness control include (multistep) spin coating, spray deposition, blade coating, and slot die coating followed by an annealing process to promote grain growth and reduce phenomena of carrier recombination and scattering at the SNC boundaries.^214,233,237,238^ Like in the case of classically grown CZTS films, selenization is sometimes performed to increase the performance of SNC-derived CZTS films too.^239^ The (partial) conversion to CZTSe shifts the absorption to longer wavelengths (given that CZTS and CZTSe have bandgaps of 1.6 and 1.0 eV, respectively) and is reported to improve carrier mobility and concentration.^240^ Note that also SNC-based cells have been reported that do not require post-deposition annealing. To that end, Korala *et al.* highlighted the central role played by SNC surface chemistry to achieve effective, uniform passivation and hence increase the values of open-circuit potential (*V*_OC_) by hundreds of mV.^241^ Cu-based multinary SNCs are also used in QDSSCs as the photoanode material.^242,243^ Already in 2014 the Kamat group reported on the size-dependent efficiency of CuInS_2_ SNC based QDSSCs, identifying a threshold size above which the PCE value dropped drastically (Fig. 11b).^244^ This cutoff was interpreted in light of a decreased charge separation achieved with narrowing bandgap. In addition, luminescent SNCs can be used to prepare light-converting layers in luminescent solar concentrators (LSCs). CuInS_2_-based SNCs are particularly suited for this type of device, since a large Stokes shift (Fig. 7c) is required for minimizing self-reabsorption over long distances (Fig. 11c).^245–247^ Core/shell QDs, gradient shells, and doping of the SNCs are all strategies that have been pursued to increase the efficiency of LSCs based on these SNCs. Moreover, Wu *et al.* recently showed that the Stokes shift can be further enhanced by dispersing the SNCs in thiol–ene polymers, reaching record PCE values of 1.36% for a 29 × 29 cm^2^ LSC device.^248^

Other optoelectronics uses of multinary Cu-based SNCs include photodetectors^256–259^ and light-emitting devices (LEDs).^125,260,261^ Regarding the latter application, the tunability of the photoluminescence of, *e.g.*, CuInS_2_-based SNCs makes them suitable for visible as well as NIR-emitting LEDs. Moreover, the SNCs can be used both as electroluminescent species^261^ or as light-converting layer in blue-emitting LEDs to achieve white light emission.^262^ However, one limitation in the use of these species in LED-based applications is the relatively broad emission spectrum, which limits the color purity achievable with these SNCs.

In a recent publication, Sachdeva *et al.* demonstrated that plasmonic CuInS_2_ SNCs are also of interest in optoelectronics due to their ability of generating hot holes under sub-bandgap (NIR) excitation.^139^ Intriguingly, when a CdS shell is grown on plasmonic CuInS_2_ SNCs, the hot holes are delocalized to the shell material, where they can be extracted for further use.^263^

##### Photocatalysis

Multinary Cu-based chalcogenides have been used for photocatalysis above all in hydrogen evolution, pollutant degradation, and CO_2_ reduction. Often, the SNCs are supported on a substrate that participates in the catalytic reaction forming a heterojunction. TiO_2_ and g-C_3_N_4_ are often the substrate of choice, with the SNCs acting as sensitizers for the substrate material. Like in the case of biomedical applications (see below), systems based on CuIn(S,Se)_2_ SNCs are the most explored ones. This is mainly because they feature a favorable band alignment to achieve an efficient S-scheme (Step-scheme) heterojunction and can extend the absorption capabilities of the substrate in the visible/NIR range. To that end, Li *et al.* reported on CuInSe_2_/(CuInSe_*x*_S_1−*x*_)_5_/CuInS_2_ core/shell/shell SNCs for photoelectrochemical hydrogen generation.^249^ The SNCs were prepared *via* cation exchange starting from CdSe/(CdSe_*x*_S_1−*x*_)_5_/CdS and then introducing in the lattice first Cu^+^, followed by In^3+^ (Fig. 11d). The resulting 1200-nm emitting SNCs were used for photo-electrochemical generation of H_2_ in the presence of Na_2_SO_3_ with photocurrent density as high as ∼4.5 mA cm^−2^. A similar concept was recently proposed by Lee *et al.* in a study aimed at understanding the formation mechanisms of CuInS_2_ SNCs *via in situ* small-angle X-ray scattering and *ex situ* XRPD and X-ray absorption.^264^ After investigation of the growth dynamics of the SNCS, the authors used core-only CuInS_2_ SNCs to prepare a photo-electrochemical device that showed a current density >8 mA cm^−2^. CuInS_2_ SNCs were also employed by Zhang *et al.* together with g-C_3_N_4_ to prepare an S-scheme heterojunction for both H_2_ evolution and pollutant (tetracycline) degradation, showing the versatility of this type of composite for photocatalysis applications.^265^ In a recent study, Yu and co-workers showcased the suitability of quaternary SNCs too for solar-to-hydrogen conversion.^250^ In the study, the authors developed a synthesis method to grow polytypic SNCs where a kesterite phase is epitaxially grown onto a wurtzite structure. Single- and double-homojunctions can be created at the single-SNC level (Fig. 11e), with double-homojunction systems featuring a better photocatalytic performance. Several other examples of similar S-scheme systems for H_2_ evolution can be found in the literature that are based on other multinary copper chalcogenides, including, *e.g.*, CuCo_2_S_4_,^266,267^ Cu–Zn–S,^268^ Cu–Sb–S.^269^ Clearly the band alignment is pivotal to ensure effective photocatalytic performance. To that end, Chai *et al.* demonstrated that the amount of Sn^2+^ and Cu^2+^ doping in Cu_2_ZnSnS_4_ nanosheets could be controlled through the synthesis temperature.^251^ An increased amount of this type of doping induced a change from p- to n-type semiconductor, and a change in the band structure and Fermi energy position. As a result, faster CO_2_ photoreduction was achieved, with reported values of 48.14 and 25.04 μmol g^−1^ h^−1^ of CO and CH_4_, respectively (Fig. 11f). While this is but a small collection of studies on the subject, the interested reader could find additional material on the subject in a dedicated review.^270^

##### Biomedicine

Multinary Cu-based SNCs are generally used in biomedicine because of their capabilities of acting as luminescent probe or for their ability to convert light into heat for photothermal applications.

Almost all the literature on photoluminescent multinary Cu-based SNCs for biomedical applications deals with CuInS_2_ and in some cases CuInSe_2_. Since the first demonstration of the use of CuInS_2_/ZnS SNCs for *in vivo* lymph node imaging in 2010 by Pons *et al.*,^271^ a plethora of publications on the biomedical use of these SNCs have seen the light. For instance, chitosan-coated CuInS_2_/ZnS SNCs were used for both cell and *in vivo* imaging by Deng *et al.*^272^ Lv *et al.* reported on pegylated phospholipid-coated CuInS_2_/ZnS SNCs as a theranostic platform capable of performing as fluorescence and photoacoustic contrast agent, while acting as photothermal and photodynamic therapy agent.^273^ CuInSe_2_/ZnS SNCs were also used by Lian *et al.* as NIR-II (1000–1350 nm) emitting nanoparticles for developing bioassays targeting circulating tumor cells (Fig. 11g).^252^ Moving from In-based and luminescent systems, CuFeS_2_-Au nanohybrids were employed by Wen *et al.* as SERS sensors for the detection of lung cancer cells and the relative biomarkers.^274^

Crucially, the toxicity of this type of SNCs started being explored too, with early reports on both cells and small animal models (*C. elegans* and mice) showing negligible toxicity due to the high chemical stability of the SNCs.^275^ More recent studies by the Dennis group introduced a more critical view on the matter, finding that the presence of a ZnS shell is critical in imparting sufficient stability to minimize – yet not completely eliminate – CuInS_2_ dissolution and hence release of toxic copper ions (Fig. 11h).^253^

Virtually any poorly- or non-luminescent, Cu-based multinary SNC can be used in light-to-heat conversion applications, given the large absorption coefficient displayed by these materials extending towards the NIR region. Indeed, SNCs made of CuFeS_2_,^276–278^ Cu_3_BiS_3_,^279^ Cu–Sb–S,^185^ Cu_3_SnS_4_,^280^ and Cu–Co–S^281^ are just some of the nanocrystals explored in this context. Often, the light-to-heat conversion capabilities are harnessed for preparing a theranostic nanosystem. To that end, Hou *et al.* showed how poly(vinylpyrrolidone)-coated Cu–Sb–S SNCs can be used simultaneously for photothermal and photodynamic therapy, while acting as photoacoustic imaging contrast agents.^185^ Yuan *et al.* also showed that CuCo_2_S_4_ SNCs have further potential as magnetic resonance contrast agents – due to the presence of Co in their composition – besides acting as effective photothermal agents, with HCE values >70%.^281^ The presence of Bi in Tween-20-coated Cu_3_BiS_3_ SNCs was instead leveraged by Liu *et al.* for computed tomography as an additional imaging modality besides photoacoustic.^279^ Of course, plasmonic SNCs are better suited for these applications, since their NIR LSPR modes can be harnessed to use optical excitation falling at different wavelengths. By the same token, Cu–Fe–S SNCs – with their NIR absorption supported by IB transitions and LSPR modes – are particularly attractive in this context.

##### Others

Other applications of these SNCs include sensing of metal species, as well as luminescence thermometry and manometry. Alayeto *et al.* recently showcased the used of bright CuInS_2_-based SNCs for selective Cd^2+^ sensing harnessing the photoluminescence enhancement induced by incorporation of the heavy metal ion in the SNC structure (Fig. 11i).^254^ Marin *et al.* prepared CuInS_2_ SNC-polymer composites capable of acting as luminescent thermometers,^123^ while Duda *et al.* applied a multimodal approach to analyse the luminescence signal of CuInS_2_/ZnS SNCs for enhanced thermal sensing performance.^282^ Aldaz-Caballero *et al.* also proposed CuInS_2_ SNCs as luminescent manometers, showing tuning the SNC size and Cu-content impacted the sensing performance of the probes (Fig. 11j).^255^ It should also be highlighted that the company UbiQD is on the market with CuInS_2_-based solutions for optimization of the solar spectrum in greenhouses. The technology relies on light absorption and conversion in specific spectral windows to maximize crop yield.^283^

## 5. Halides

### 5.1. General considerations, structure, and optical properties

**Fig. 12:**
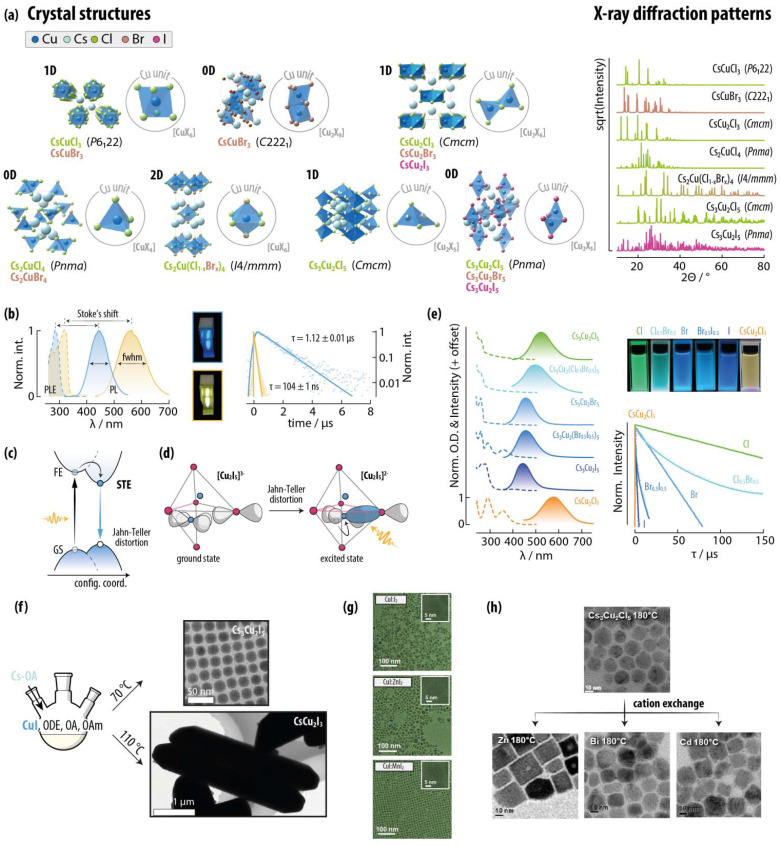
Caesium copper halides as representative of copper halide SNCs. (a) Unit cell, copper cluster geometry, and simulated XRPD patterns of the most common compositions reported for Cs–Cu–X (where X = Cl, Br, I). (b) Absorption (dashed lines) and emission (solid lines) spectra of Cs_3_Cu_2_I_5_ (blue) and CsCu_2_I_3_ (yellow), alongside a photo of the dispersion under UV excitation, and the corresponding photoluminescence decay curves. Adapted with permission from ref. 286, Copyright 2019, American Chemical Society. (c) The emission stems from a self-trapped exciton. (d) Representation of the Jahn–Teller distortion that occurs after excitation in Cs_3_Cu_2_I_5_. Adapted with permission from ref. 287, Copyright 2025, Springer. (e) Absorption and emission spectra, photoluminescence decay curves, and phot under UV excitation of a series of alloyed Cs_3_Cu_2_X_5_ SNCs, alongside CsCu_2_Cl_3_ SNCs. Adapted with permission from ref. 288, Copyright 2020, American Chemical Society. (f) Scheme of the synthesis protocol for the synthesis of Cs_3_Cu_2_I_5_ SNCs or CsCu_2_I_3_ microcrystals, alongside the TEM images of the respective materials. Adapted with permission from ref. 284, Copyright 2019, John Wiley and Sons. (g) TEM images of Cs_3_Cu_2_I_5_ SNCs obtained using different halide additives, showing optimized morphology and narrow size distribution in the presence of MnI_2_. Adapted with permission from ref. 289, Copyright 2022, American Chemical Society. (h) TEM images of parent Cs_3_Cu_2_Cl_5_ synthesized at 180 °C and used as sacrificial species for cation exchange with Zn, Bi, and Cd. The TEM images of the SNCs obtained from the cation exchange procedures are reported in the bottom row. Adapted with permission from ref. 290, Copyright 2023, American Chemical Society.

The research on ternary copper halides SNCs has mainly stemmed from the research on halide perovskites, in pursuit of less toxic and more stable nanomaterials that are equally performing in optical and optoelectronic applications. This family of materials is the most recent development in the research of Cu-based SNCs, with the seminal paper on Cs_3_Cu_2_I_5_ and CsCu_2_I_3_ SNCs published by Cheng *et al.* in 2019.^284^ The most common stoichiometries encountered for these materials are ACuX_3_, ACu_2_X_3_, A_2_CuX_4_, A_3_Cu_2_X_5_ – where A = K, Rb, Cs and X = Cl, Br, I.^14^ These materials crystallize mainly into 0D or 1D structures,^285^ where Cu-containing clusters are individually distributed (0D) or arranged along ribbons (1D) interspersed by A^+^ ions. Given that the SNCs that can be found in the literature are mainly Cs-based, here we report on those materials specifically (Fig. 12).

In the CuCsX_3_ family, CsCuCl_3_ crystallizes in a chiral structure, in either *P*6_1_22 (right-handed) or *P*6_5_22 (left-handed) space group with octahedral [CuCl_6_] units.^291,292^ Intriguingly, also CsCuBr_3_ takes up a crystal structure with *P*6_5_22 space group above 420 K, while the thermodynamically stable phase is orthorhombic (*C*222_1_), with dimeric units of face-sharing [CuBr_6_] octahedra. The reported bandgap values of these materials vary substantially between reports. CsCuCl_3_ has an indirect bandgap (1.9–2.6 eV), while CsCuBr_3_ has a somewhat wider, direct bandgap (2.63 eV). There is currently no report on CsCuI_3_.

The three ternary halides of the CsCu_2_X_3_ family all crystallize in the *Cmcm* (or possibly *Pbnm*) space group, featuring tetrahedral [CuX_4_] units sharing edges to form 1D chains along the *c*-axis.^293,294^ At room temperature, reported bandgaps are in the 3.0–3.75 eV range and direct in nature.^295,296^ Cs_2_CuCl_4_ and Cs_2_CuBr_4_ are found to crystallize mainly in the orthorhombic *Pnma* space group where Cu^2+^ resides in distorted tetrahedra, although mixed phases with Cs_2_Cu(Cl_1−*x*_Br_*x*_)_4_ at specific growth conditions can take up a tetragonal *I*4/*mmm* structure.^297^ No instances of Cs_2_CuI_4_ are reported in the literature.

The Cs_3_Cu_2_X_5_ family (with bulk bandgaps in the 3.9–4.3 eV range) is of particular interest for the preparation of SNCs,^298^ since these semiconductors generally feature strong emission intensity (reaching PLQY ≈ 1), coupled with efficient light absorption capabilities (albeit values of absorption coefficient are not reported). This family of materials tends to crystallize in the orthorhombic *Pnma* space group where [Cu_2_X_5_]^3−^ dimers are separated by A^+^ ions to give a 0D structure. Cs_3_Cu_2_Cl_5_ has been shown to take up also the *Cmcm* space group with two non-equivalent Cu^+^ ions disordered over two tetrahedral sites to form 1D structures.^288,299^ Intriguingly, doping can be used to enhance the optical properties of these materials. To that end, Chen *et al.* demonstrated that Cs_3_Cu_2_I_5_ SNCs can be doped with Rb^+^ – albeit only with an efficiency close to 20%. This process yielded wider band gap and higher PLQY (reaching almost unity), owing to the distortion induced by Rb^+^ substitution at one of the Cs^+^ sites.^300^ An overview of the structures, dimensionality, and cluster geometry of Cs–Cu–X – which are the most studied materials of this type – is reported in Fig. 12a.

The luminescence of these materials is characterized by the presence of strongly localized self-trapped excitons (STEs). STEs arise when there is strong electron–phonon coupling, and the electron–hole pair is strongly confined due to lattice distortions. Indeed, photoexcitation in these materials also results in a marked Jahn–Teller effect due to the change from Cu(i) 3d^10^ to Cu(ii) 3d^9^ electronic configuration in tetrahedral coordination,^287^ which stabilizes the STE (Fig. 12c and d). Note that depending on the dimensionality of the structure, the excitons are localized over individual Cu-containing clusters (0D), or along ribbons (1D) and planes (2D) of clusters separated by A^+^ ions. The presence of STEs yields in these materials broad emission peaks,^286^ lifetimes up to 10s of microseconds, and marked Stokes shifts – at times larger than 1.5 eV (Fig. 12b).^293^

Much alike halide perovskites, a strategy employed for fine-tuning the optical properties of these SNCs is mixing different halides to obtain alloyed SNCs (Fig. 12e).^288,301,302^ This is a particularly successful strategy for Cs_3_Cu_2_X_5_, where all the halides crystallize in the same structure. Another important feature of these materials is the relatively low charge mobility, owing to rather flat electronic bands (resulting in high effective mass).

The most common compositions synthesized at the nanoscale include CsCu_2_X_3_ and Cs_3_Cu_2_X_5_, where copper is in its +1 oxidation state.

### 5.2. Synthesis

Synthesis protocols for the preparation of SNCs made of these materials take clear inspiration from the methods developed for halide perovskites. Specifically, the synthesis approach generally entails the rapid injection of Cs^+^ precursors in a Cu + X solution to induce controlled precipitation of the SNCs in the presence of ligands such as fatty acids and amines. In the first report on this family of materials, selective access to SNCs of CsCu_2_I_3_ or Cs_3_Cu_2_I_5_ was achieved by controlling the injection temperature of Cs-oleate in a CuI, ODE, OA, and OAm mixture (Fig. 12f).^284^ Later, Chatterjee *et al.* reported on the preparation of both quasi-spherical SNCs and rod-shaped Cs_3_Cu_2_I_5_ microcrystals using respectively a controlled precipitation method at low temperature (25 °C) and a hot injection protocol (130 °C).^303^ In both cases, the injection of the Cs^+^ precursor is followed by rapid quenching in ice to prevent further crystal growth. Park *et al.* demonstrated that in this type of reaction the presence of excess Cu-oleate is pivotal to obtain well-formed and passivated cubic SNCs, and identified MnI_2_ as a better iodide additive compared to I_2_ or ZnI_2_ (Fig. 12g).^289^ The authors attributed this observation to the capability of Mn^2+^ to favor Cu-oleate from CuI in the presence of OA. This is the result of the catalytic capabilities of manganese, driven by the ion's multiple possible oxidation states. Moreover, Le *et al.* identified the length of the alkyl chain of the organic ligands (acid-amine pair) as a key parameter in controlling the size and morphology of Cs_3_Cu_2_Br_5_ SNCs prepared *via* hot injection.^304^ In the same study, alloying with different halides (Cl and I) was also achieved to fine-tune the SNC optical properties.

Zhang *et al.* showed that the reverse approach can also be used, and they achieved the preparation of Cs_3_Cu_2_I_5_ SNCs by injecting a CuI solution in a mixture of Cs_2_CO_3_, ODE, OA, and OAm.^305^ These types of reactions can also be streamlined into continuous flow systems – as demonstrated by Urban and co-workers^306^ − for enhanced reproducibility and tunability of the SNC size and composition.

Intriguingly, Kwon *et al.* proposed the use of Cs_3_Cu_2_Cl_5_ SNCs as sacrificial seeds for conducting cation exchange processes to access Cs_2_ZnCl_4_, Cs_3_BiCl_6_, and CsCdCl_3_ SNCs (Fig. 12h).^290^ The structural changes (also accompanied by morphological variations) that cation exchange induced are such that further exchange with Cu^+^ leads to SNCs of a material different from the starting one: CsCu_2_Cl_3_. Very recently, Rogach and co-workers also demonstrated that CsPbBr_3_-Cs_3_Cu_2_Br_5_ nano-heterostructures can be prepared *via* epitaxial growth. These composite nanosystems show a pseudo-type II band alignment and offer an attractive platform to prepare heterojunctions with fine-tuned band architectures.^307^

### 5.3. Applications

Given the more recent history of SNCs with these compositions, examples of their applications are relatively limited. Yet, there are promising indications of the suitability of Cu-based halide SNCs in some specific fields. The attractiveness of these SNCs compared to, *e.g.*, Pb-based perovskite SNCs is generally attributed to the higher environment friendliness coupled with better stability – often imparted through careful doping strategies.

#### 5.3.1. X-ray imaging

**Fig. 13:**
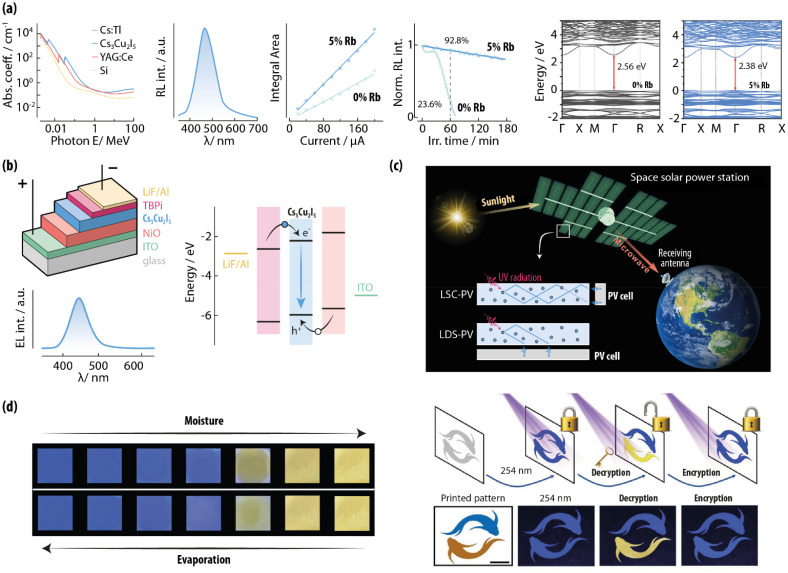
Caesium copper halides as representative of copper halide SNCs. (a) Potential of Cs_3_Cu_2_I_5_ SNCs for the preparation of scintillation devices. From left to right: X-ray absorption coefficient for Cs_3_Cu_2_I_5_ SNCs compared to other materials used for scintillation, emission spectrum of Cs_3_Cu_2_I_5_:Rb(5%) SNCs under X-ray excitation, the integrated area of undoped and 5% Rb doped SNCs as a function of driving voltage of the X-ray source, stability study under continuous X-ray irradiation, and band structure of the undoped and doped SNCs. Adapted with Permission from ref. 300, Copyright 2025, John Wiley and Sons. (b) Scheme of the optoelectronic device used as blue LED with a Cs_3_Cu_2_I_5_ SNC emissive layer, alongside the electroluminescence spectrum of the LED, and the approximate band alignment of the various layers. Adapted with Permission from ref. 298, Copyright 2020, American Chemical Society. (c) Scheme showing the envisioned applicability in space power stations of luminescent solar concentrators containing Cs_3_Cu_2_Cl_5_ SNCs. Adapted with permission from ref. 313, Copyright 2023, American Chemical Society. (d) Change of colour in an anticounterfeiting ink driven by the moisture-driven conversion between Cs_3_Cu_2_I_5_ and CsCu_2_I_3_ SNCs. An example of the use of this ink for data encryption is also shown. Adapted with permission from ref. 305, Copyright 2021, John Wiley and Sons.

The scintillation capabilities of CsCu_2_X_3_ and Cs_3_Cu_2_X_5_ SNCs have been fully embraced by the research community over the past five years. Examples of X-ray stimulated luminescence (*i.e.*, radioluminescence) have been reported for SNCs of CsCu_2_I_3_,^308^ Cs_3_Cu_2_Cl_5_,^309,310^ and Cs_3_Cu_2_I_5._^300,311,312^ In fact, the X-ray absorption coefficient of these materials is on par with CsI:Tl (Fig. 13a) – a staple scintillator material. The high radioluminescence efficiency is supported by high PLQY values at times close to 90%. Very recently Chen *et al.* showed that doping with Rb^+^ is an effective strategy to increase both the PLQY and the stability under continuous X-ray irradiation of Cs_3_Cu_2_I_5_ SNCs.^300^*Via* simulations, the authors identified doping of Rb at a Cs site as the most probable doping mechanism, which resulted in reduced electron–phonon coupling and overall suppressed non-radiative recombination pathways. In the various studies mentioned above, the preparation of X-ray detection screens was achieved by depositing the SNCs on various surfaces, including paper, or incorporating them into a polymeric matrix to prepare standalone films. The attractiveness of these strategies resides in the easy processability of SNC-based films and the lack of highly toxic thallium in the scintillator.

#### 5.3.2. Light emitting devices

Cs_3_Cu_2_X_5_ SNCs have been used in LEDs both as electroluminescent material^314^ and to prepare light conversion layers. To that end, in one of the first reports on this use of Cs_3_Cu_2_X_5_ SNCs, Wang *et al.* prepared a blue LED with half-lifetime >100 h and an external quantum efficiency of 1.12% (Fig. 13b).^298^ Therein, the SNC layer acted as the electroluminescent element, showing good resistance to heating to >80 °C and storage in air for >30 days. The groups of Park^315^ and Ye,^316^ instead used respectively Mn-doped and Mg-doped Cs_3_Cu_2_X_5_ SNCs as light converting layers in LEDs based on UV-emitting chips. Mn^2+^ introduced additional radiative relaxation pathways (through d–d transitions) that extended the color gamut of the SNCs. The resulting LED showed optical output powers on par with commercial white LEDs, yet a better spectral overlap (88.8 *vs.* 32.4%) with the solar spectrum. Mg^+^ doping was instead leveraged to increase the PLQY of the SNCs, while also improving their stability when exposed to high temperatures and high-humidity environments.

#### 5.3.3. Others

Other proposed applications for these nanomaterials include photovoltaic devices, photodetectors, photocatalysis, luminescent inks, and sensing. As mentioned above, the mobility of the charge carriers in these materials is relatively low, as evinced by the flat electronic band structure (Fig. 13a). Therefore, the performance of SNCs with these compositions is limited in applications that require fast charge separation. Still, some examples are reported in the literature. In that vein, Daliran *et al.* developed a mesoporous metal organic framework (MOF) incorporating CsCu_2_I_3_ SNCs as a composite photocatalyst.^317^ The *in situ* growth within the MOF pores granted control over the SNC size and imparted stability to the halides. The authors demonstrated the suitability of the system to catalyze cycloaddition and photo-oxidation/Knoevenagel condensation cascade reactions under visible LED illumination (1 W, 32 000 lux), identifying the presence of multiple Lewis acid sites (Fe^3+^, Zr^4+^, Cu^+^, and Cs^+^) as a key element.

Cs_3_Cu_2_Cl_5_^313^ and Cs_3_Cu_2_I_5_^318^ SNCs were also explored in photovoltaic devices for their UV harvesting capabilities. Albeit a large portion of UV light with wavelength shorter than 300 nm does not reach Earth surface due to ozone absorption, the interest around photovoltaic devices working in that spectral range is underpinned by their possible use in space power stations (Fig. 13c). To that end, Wang *et al.* showed that Cs_3_Cu_2_Cl_5_ SNCs are suitable for LSC and as luminescent downshifting (LDS) layers^313^ when incorporated in a siloxane polymer (PDMS), owing to the large Stokes shift – limiting re-absorption – and PLQY > 80%. A similar concept was behind the work of Liu *et al.*, who boosted the efficiency of Si photodetectors in the UV through the implementation of a downshifting Cs_3_Cu_2_Cl_5_ SNC polymeric layer.^319^

Given the brightness of these SNCs, it is unsurprising that preparation of luminescent inks has also been reported.^320^ Some of the anticounterfeiting strategies are multi-level in nature, such as the moisture-sensitive inks developed by Zhang *et al.* (Fig. 13d),^305^ Wang *et al.*,^321^ and Feng *et al.*^322^

Sensing is yet another application field explored for these nanomaterials, with a report on chemical sensing of tebuconazole (TEB, a fungicide).^323^ The sensing mechanism is based on photoluminescence quenching of 1,10-phenanthroline-passivated Cs_3_Cu_2_I_5_ SNCs, which is induced by TEB-induced aggregation in a concentration-dependent fashion.

## 6. Conclusions

We have reviewed the recent advances in the field of Cu-based semiconductor nanocrystals (SNCs) and their optical properties. Specifically, these nanomaterials exhibit photoluminescence and plasmonic properties, making them attractive for applications such as photovoltaics, lighting, sensing, photocatalysis, and X-ray imaging.

Of the three families discussed herein, pnictogenides and chalcogenides display stark similarities, featuring robustness against stoichiometry deviations and tunability of the optical properties depending on the composition of the material. Specifically, SNCs with compositions characterized by partially-filled valence bands display quasi-free holes that can support localized plasmonic resonances. A unique case is represented by CuFeE_2_ SNCs, which support both localized plasmonic resonances and dielectric resonance modes arising from Fe 3d intrabandgap states. Photoluminescence in these families of SNCs stems instead from different mechanisms. For CuInE_2_ and CuGaE_2_ SNCs – the more explored compositions in pursuit of photoluminescence – the consensus in the community points to a free-electron-to-bound-hole as the most probable radiative recombination mechanism.

The third family, Cu-based halide SNCs, has markedly different properties. The main interest in them stems from an efficient photoluminescence, which makes these SNCs an attractive, less toxic alternative to halide perovskites. The crystal structure of Cu-based halide SNCs is characterized by Cu-halide clusters arranged in 0D, 1D, or 2D patterns where self-trapped excitons (STEs) get localized.

Despite the exciting optical features of Cu-based SNCs, our increased understanding of structure-properties relationships, and refinement of the synthesis protocols, several outstanding challenges and opportunities lie ahead in this field.

For starters, in the vast body of literature on Cu-based SNCs, some compositions are much less explored than others. This is particularly the case of nitrides and tellurides. Nitride SNCs are generally challenging to synthesize *via* colloidal chemistry, and Cu_3_N is indeed the more synthetically-accessible composition in the family.^324^ The research on nitride SNCs is also being driven by the interest in the plasmonic properties of these materials.^325^ The identification of precursors that are safe to handle and with controlled reactivity, as well as alternative synthesis routes would certainly stir up interest for Cu-based nitride SNCs, allowing a more thorough study of their size- and morphology-dependent optical properties. The scarcity of tellurium-containing precursors and ligands is behind the reduced number of reports on Cu-based SNCs containing this chalcogen. In fact, tellurium chemistry is characterized by its malodorous and toxicity, as well as its instability and sensitivity to air and light, making this element less than appealing to work with. Yet tellurium is unique in that it has a diffuse electron density and shows increased spin–orbit coupling. One direct consequence is the reduced bandgap often featured by tellurides compared to sulfides and selenides, which results in more red-shifted optical absorption and photoluminescence. The electronic band structure is also modulated, resulting in tunable electronic properties, such as carrier mobility. As such, increased effort in the preparation of telluride Cu-based SNCs could lead to nanomaterials with uncommon, attractive optical properties.

Focusing on chalcogenides, phase transitions upon exposure to even mild temperatures and during storage are a concern in Cu–E SNCs. This intrinsic phase instability arises from the multiple polymorphs observed in Cu–E and the low diffusion barrier in these materials. Structural changes alter electronic/optical properties, hence reducing the credibility of technological solutions based on Cu–E SNCs. A solution to this issue might require a multipronged approach to obtain new, more complex Cu–E SNCs with minimized ion diffusion (*e.g.*, *via* doping or alloying) and resistance to surface oxidation (*e.g.*, through the growth of core/shell nanostructures). Future research aimed at understanding phase transitions in these nanomaterials at the atomic level and, thus, guiding the design of stable Cu–E SNCs could yeild results with tremendous technological impact.

Cu-based halide SNCs are generally considered more stable than, *e.g.*, Pb-based halide perovskite SNCs; however, their long-term stability remains a concern. In these materials, exposure to air and/or moisture can lead to surface oxidation, mainly driven by the redox chemistry of the Cu(i)/Cu(ii) system. It is conceivable that strategies recently developed to increase the surface stability of halide perovskites can be applied to these Cu-based SNCs, such as the use of multidentate ligands^326^ and shell formation with inorganic materials.^327^ Partial substitution of Cu(i) for other metals, *e.g.*, Ag(i), that are less readily oxidized can also improve stability – though inducing changes in the optical and electronic properties. Additionally, the soft crystal structure of halides (extensively discussed in halide perovskites^328^) leads to substantial anion migration within the lattice, which can adversely affect the stability of optoelectronic devices. Strategies used to limit ion migration in Pb-halide perovskites are expected to be similarly effective in Cu-based systems. These include defect engineering *via* aliovalent element doping and tuning of metal-halogen bond strength.^329^ The former could be achieved *via* interstitial doping with ions such as Ca^2+^ and Ba^2+^ to increase the activation energy for halide migration. The latter could be accomplished by partial cation substitution of Cu(i) with Au(i), which forms a stronger metal–halide bond.

The relatively low mobility of charge carriers in Cu-based halide SNCs poses instead major limitations to their use in applications that require efficient charge extraction, such as photocatalysis or photoelectronic devices. An exciting avenue for future research is the identification of approaches to modify the electron structure of the material to modulate the effective mass of the charge carriers, including doping and strain engineering.

The preparation of complex heterostructures is another approach expected to yield materials with a unique combination of optical and electronic properties. For example, plasmonic and luminescent properties can be achieved in a single heterostructure based completely on copper. In addition, more effective carrier extraction can be obtained by modulating the electronic band alignment in multiple materials in contact within the same heterostructure. Cation (and anion) exchange strategies, as well as controlled epitaxial growth, are ideal tools to achieve such structures. However, the ultimate success of these sophisticated structures remains contingent upon resolving the stability issues mentioned above.

To conclude, the technological relevance – either already validated and exploited or just postulated – of Cu-based SNCs is expected to drive further research on these nanomaterials. As such, we believe that the future of this field still has plenty to offer chemists, physicists, materials scientists, and engineers alike. We hope that more of the systems proposed as proof-of-concept would move towards large-scale implementation and on the market, where they can benefit society for a brighter future.

## Author contributions

R. M. contributed to the conceptualization, funding acquisition, visualization, and writing of the original draft. L. V. B. and P. C. contributed to reviewing and editing. P. C. also contributed to data visualization.

## Conflicts of interest

There are no conflicts to declare.

## Data Availability

No primary research results, software or code have been included and no new data were generated or analysed as part of this review.
